# The efficacy and safety of intraocular anti-VEGF injections versus anti-VEGF combined with steroids or steroid monotherapy for macular edema secondary to retinal vein occlusion: a systematic review and meta-analysis of randomized controlled trials

**DOI:** 10.3389/fmed.2025.1727801

**Published:** 2026-01-12

**Authors:** Han Cai, Miao Tian, Zhilong Huang, Binglong Wang

**Affiliations:** 1 Ophthalmology Centre, Renmin Hospital of Wuhan University, Wuhan, Hubei, China; 2 Guangzhou Medical University, Guangzhou, Guangdong, China; 3 School of Health Policy and Management Chinese Academy of Medical Sciences and Peking Union Medical College, Beijing, China

**Keywords:** macular edema, meta-analysis, randomized controlled clinical trials, retinal vein occlusion, steroids, vascular endothelial growth factor

## Abstract

**Objective:**

The study performed a systematic review and meta-analysis of randomized controlled trials (RCTs) assessing the efficacy and safety of intraocular injections of anti-vascular endothelial growth factor (VEGF) monotherapy versus steroid monotherapy or anti-VEGF combined with steroids for macular edema (ME) secondary to retinal vein occlusion (RVO).

**Materials and methods:**

We systematically searched PubMed, Embase, Web of Science, and the Cochrane Library from database inception to 10 August 2025 for randomized controlled trials (RCTs) comparing anti-VEGF or steroid monotherapy with their combination.

**Results:**

A total of 22 randomized controlled trials (RCTs) were included. Of these, 13 trials (mean difference, −43.21, 95% CI, −76.82 to −9.60, *p* = 0.01) compared anti-VEGF monotherapy with steroid monotherapy or combination therapy, and the results showed that anti-VEGF monotherapy was more effective in improving central macular thickness (CMT). Furthermore, seven trials used the Early Treatment Diabetic Retinopathy Study (ETDRS) letters to record changes in best-corrected visual acuity (BCVA). The pooled results (mean difference, 5.72, 95% CI, 1.82 to 9.61, *p* = 0.004) indicated that anti-VEGF monotherapy was more effective compared to the other two treatments. A total of 10 trials used the standard logarithm of the minimum angle of resolution (logMAR) visual acuity (VA) chart to assess best-corrected visual acuity. The pooled results (mean difference, 0.01, 95% CI, −0.09 to 0.12, *p* = 0.80) indicated that anti-VEGF monotherapy has no significant advantage over combination therapy or steroid drugs. A total of eight adverse events were included in the analysis: cataract, conjunctival hemorrhage, eye pain, intraocular pressure (IOP), increased lacrimation, macular edema, ocular hypertension, and reduced visual acuity. Compared to steroid monotherapy, anti-VEGF monotherapy can reduce the incidence of cataract, elevated intraocular pressure, ocular hypertension, and reduced visual acuity. In addition, compared to combination therapy, anti-VEGF monotherapy can reduce the occurrence of ocular hypertension.

**Conclusion:**

This meta-analysis indicates that monotherapy with anti-VEGF drugs is more effective than the other two treatment methods in reducing CMT in patients with retinal vein occlusion. Regarding the ETDRS scores, anti-VEGF monotherapy was better than the other two treatments.

**Systematic review registration:**

https://www.crd.york.ac.uk/PROSPERO/view/CRD420251146756, CRD420251146756.

## 1 Introduction

Retinal vein occlusion (RVO), with a prevalence of 5.2 per 1,000 individuals, is the second most common cause of visual loss among retinal vascular diseases, following diabetic retinopathy (1). RVO affects approximately 1–2% of individuals aged 40 years and older and contributes substantially to visual impairment and disability (2, 3). Currently, there are approximately 16 million individuals diagnosed with RVO globally (4). Clinically observed subtypes of RVO primarily include central retinal vein occlusion (CRVO), branch retinal vein occlusion (BRVO), and hemiretinal vein occlusion—all of which can be further categorized into ischemic or non-ischemic subtypes based on the severity of vascular occlusion. Most cases of CRVO and 5–15% of patients with BRVO develop macular edema (ME) in the early stage of disease progression (5). As the most common complication of RVO, ME is a key contributor to severe visual impairment (1, 5); prolonged ME may induce permanent structural damage to the retina, ultimately leading to irreversible vision loss (6).

Historically, therapeutic options for RVO-associated macular edema (ME) were limited. Prior to the era of intravitreal therapy, macular grid laser photocoagulation served as the gold standard for the treatment of ME secondary to branch retinal vein occlusion (BRVO) (7). The Branch Vein Occlusion Study (BVOS) reported superior visual outcomes in eyes with visual acuity (VA) worse than 20/40 that were randomized to grid laser therapy versus sham treatment (8, 9). However, this approach is not suitable for central retinal vein occlusion (CRVO), primarily because CRVO is often associated with more extensive retinal ischemia, and laser treatment fails to effectively reduce vascular leakage in such cases (10). The efficacy of the intravitreal dexamethasone implant (Ozurdex) for ME due to both CRVO and BRVO was evaluated in the GENEVA study (9). The results from this trial showed that the Ozurdex implant group exhibited a higher proportion of patients (both with CRVO and BRVO) achieving a ≥ 15-letter improvement in VA at 90 days, along with a lower proportion of patients experiencing a ≥ 15-letter VA loss, compared to the sham group. In addition, the SCORE-CRVO study demonstrated that a specific dose of intravitreal triamcinolone confers therapeutic benefits for CRVO-induced ME (11).

Currently, anti-vascular endothelial growth factor (anti-VEGF) agents have demonstrated remarkable clinical efficacy and have been established as the first-line therapy for RVO-associated ME (4, 12–15). The pathophysiological mechanism underlying RVO-associated ME involves increased release of inflammatory cytokines and upregulation of VEGF, which disrupt the blood–retinal barrier (BRB) and alter vascular permeability—ultimately leading to ME development. In turn, prolonged ME may induce retinal apoptosis and fibrosis, resulting in irreversible visual impairment (6, 16). Anti-VEGF agents exert therapeutic effects by inhibiting VEGF-mediated angiogenesis and vascular leakage, thereby promoting ME resolution (17, 18). Multiple randomized controlled trials (RCTs) have provided evidence of significant visual and anatomical improvements in patients receiving intravitreal anti-VEGF injections (19–22). For instance, a real-world study involving 135 eyes reported a 14-letter VA improvement at 2 years, with a median of seven injections administered during this period (23). The LEAVO study compared the efficacy of ranibizumab, aflibercept, and bevacizumab for the management of CRVO-related ME (24). The results indicated that ranibizumab was non-inferior to aflibercept, while the aflibercept group required fewer injections. However, no conclusive evidence was obtained regarding whether bevacizumab was inferior to ranibizumab. Another real-world study involving221 eyes demonstrated the sustained long-term efficacy of anti-VEGF therapy: VA improvements (14.8 letters in BRVO eyes and 14.4 letters in CRVO eyes) were maintained over 8 years (25). Collectively, these findings confirm the substantial clinical benefits of anti-VEGF agents in the treatment of RVO-associated ME.

In recent years, an increasing number of meta-analyses have examined the efficacy of different therapeutic strategies for RVO (26–30). However, these studies had notable limitations: not all included literature consisted of randomized controlled trials (RCTs)—a critical design for evidence grading in therapeutic research—and the sample size of the included studies was relatively small. Therefore, we conducted an updated meta-analysis to systematically evaluate the safety and efficacy of intraocular injections of anti-VEGF monotherapy versus steroid monotherapy or anti-VEGF combined with steroids for macular edema secondary to retinal vein occlusion (RVO).

## 2 Materials and methods

### 2.1 Search strategy

The present meta-analysis was conducted in accordance with the 2020 guidelines established by the Preferred Reporting Items for Systematic Reviews and Meta-Analyses (PRISMA) (31). The meta-analysis has been registered with PROSPERO under registration number CRDCRD420251146756. A comprehensive search was performed across four databases—PubMed, Embase, Web of Science, and the Cochrane Library—for literature published up to 10 August 2025. The search approach adhered to the PICOS framework and incorporated a combination of MeSH terms and free-text keywords.

The specific search strategy used was as follows: “Retinal Vein Occlusion “AND “Vascular Endothelial Growth Factor “AND “Steroids “AND “Macular Edema “AND “Randomized Controlled Trials.” A comprehensive overview of the search record is provided in the Supplementary material.

### 2.2 Inclusion and exclusion criteria

The inclusion criteria were as follows: (1) Studies that included patients diagnosed with RVO-related macular edema; (2) studies in which patients in the intervention group received corticosteroids (glucocorticoid, steroids, Ozurdex, Yutiq, triamcinolone acetonide, and dexamethasone) and intravitreal anti-VEGF drugs (bevacizumab, ranibizumab, aflibercept, pegaptanib, Avastin, conbercept, and brolucizumab); (3) studies in which patients in the controlled group received corticosteroids or anti-VEGF drugs; (4) studies that reported changes in best-corrected visual acuity (BCVA) and central macular thickness (CMT); and (5) studies that were RCTs.

The exclusion criteria were as follows: (1) duplicate patient cohorts; (2) other types of articles, such as case reports, publications, theses, letters, comments, reviews, meta-analyses, editorials, and protocols; (3) studies not relevant to the research question; and (4) studies that did not report changes in BCVA and CMT.

### 2.3 Selection of articles

The literature selection process, including the removal of duplicate entries, was conducted using EndNote (Version 20; Clarivate Analytics). The preliminary search was conducted by two independent reviewers who removed duplicate records, evaluated titles and abstracts for relevance, and classified each study as either included or excluded. For trials that did not report changes in BCVA and CMT in the full text, Supplementary materials were examined to extract the relevant data. In addition, using clinical trial registration identifiers (e.g., NCT numbers) provided in the publications, the ClinicalTrials.gov database was queried to ascertain whether changes in BCVA and CMT were reported. Discrepancies were resolved by consensus. A third author of the review would assume the role of an arbiter in the absence of unanimity.

### 2.4 Data extraction

Data were independently extracted by two reviewers. The extracted data included the following: (1) Basic information of the study, including NCT number, study design, and sample size; (2) baseline characteristics of study participants, including number of patients, age, and administered drugs; and (3) changes in BCVA and CMT. Discrepancies were resolved by consulting a third investigator.

### 2.5 Quality assessment

A total of two independent reviewers performed the quality assessment of the included trials. In this study, the risk of bias in the RCTs was assessed using the Cochrane risk-of-bias tool for randomized trials. The tool assesses seven domains: random sequence generation (selection bias), allocation concealment (selection bias), blinding of participants and personnel (performance bias), blinding of outcome assessment (detection bias), incomplete outcome data (attrition bias), selective reporting (reporting bias), and other sources of bias. In the event of any inconsistencies, disagreements were resolved through collective deliberation.

### 2.6 Statistical analysis

The analyses were conducted using Cochrane’s Review Manager 5.3, R statistical software (version 4.5.1,), and GRADEprofiler. The comparison of continuous variables was performed using the weighted mean difference (WMD) with a 95% confidence interval (CI). The risk ratio (RR) with a 95% CI was used to compare binary variables. Medians and interquartile ranges of continuous data were converted to means and standard deviations. Statistical heterogeneity among the included studies was assessed using the Cochrane’s Q test and the I^2^ index. Random effects models, employing the DerSimonian–Laird method, were utilized in the absence of considerable heterogeneity (Q tests, *p* < 0.05; I^2^ > 50%); otherwise, fixed effects models using the Mantel–Haenszel method were applied.

Sensitivity analyses were performed by sequentially excluding eligible trials one at a time. Publication bias was evaluated visually using funnel plots. The certainty of evidence for each outcome in the study was evaluated using the Grading of Recommendations, Assessment, Development, and Evaluations (GRADE) framework, which considers risk of bias, imprecision, inconsistency, indirectness, and publication bias in the included studies. *p*-values of <0.05 were considered statistically significant.

## 3 Results

### 3.1 Search results

**Figure 1:**
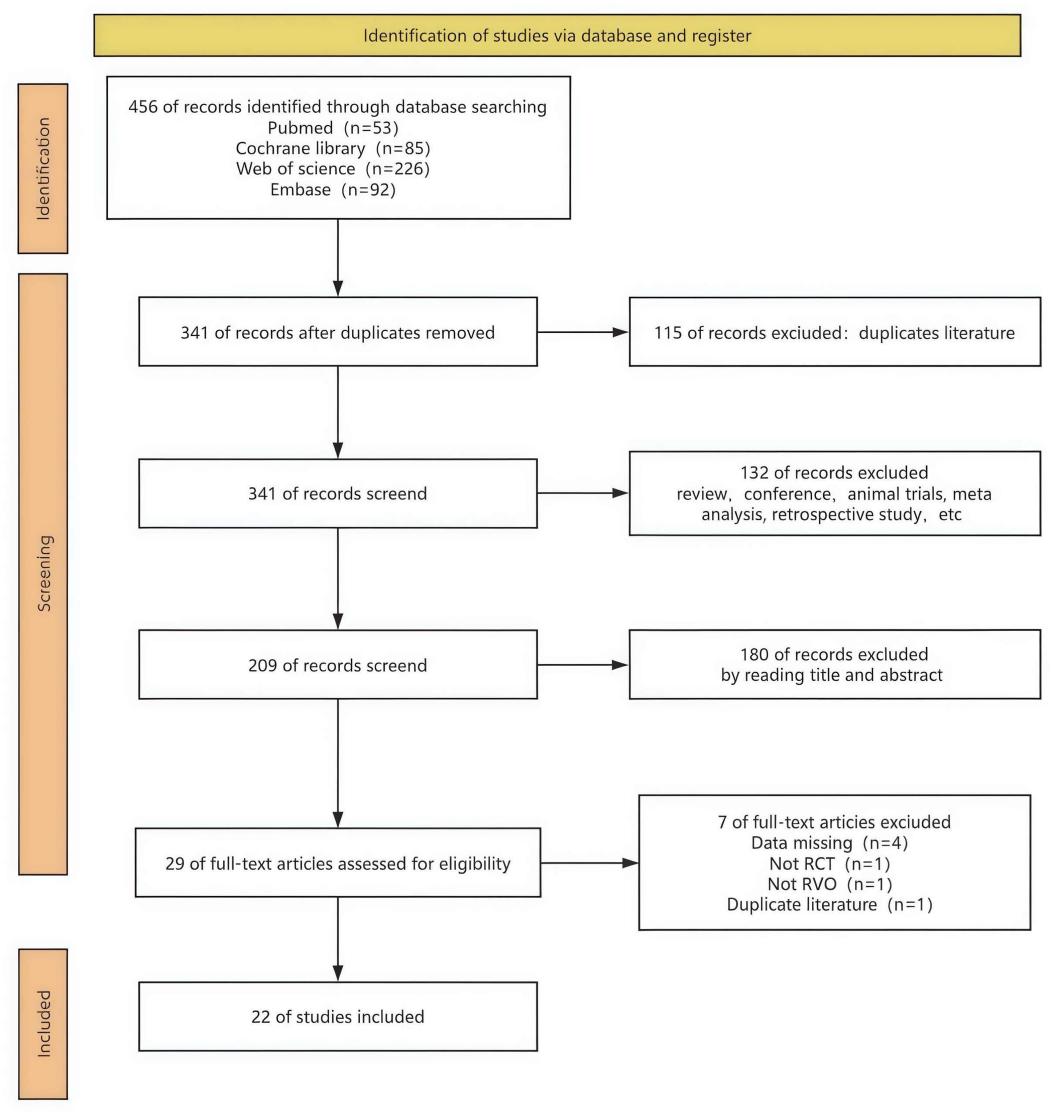
Flowchart of literature search strategies.

Figure 1 illustrates the process of selecting and incorporating literature. A total of 456 studies were initially identified. After removing 115 duplicates, primarily through the automatic screening function of the software, 341 articles remained. After excluding other literature types, such as reviews, animal trials, meta-analyses, retrospective studies, and single-case studies, 132 publications were deemed irrelevant and were excluded. Then, 209 articles were manually excluded based on titles and abstracts. Finally, after reviewing the full texts, examining Supplementary materials, and verifying trial registration numbers (e.g., NCT), a total of 22 trials were deemed eligible.

### 3.2 Patient characteristics

This meta-analysis included 22 RCTs. A total of 2,221 patients were enrolled: 844 in the corticosteroids group, 1,080 in the anti-VEGF group, and 297 in the combination therapy group. Among these trials, 11 included patients with Branch Retinal Vein Occlusion (BRVO), eight included patients with Central Retinal Vein Occlusion (CRVO), and four included patients with Retinal Vein Occlusion (RVO). The anti-VEGF agents included bevacizumab, ranibizumab, aflibercept, and conbercept. Detailed baseline characteristics of the trials and participants are presented in Table 1.

**Table 1:** Characteristics of the included studies and patients.

Author, year	NCT	No. of participants	Disease	Steroids/number	Anti-VEGF/number	Combination therapy/number
Bandello, 2018 (42)	NCT01427751	307	BRVO	DEX implant 154	Ranibizumab 153	NA
Cai, 2024 (43)	ChiCTR2400080048	44	RVO	NA	Ranibizumab 23	Ranibizumab+Dexamethason 21
Campochiaro, 2018 (44)	NCT02303184	46	RVO	NA	Aflibercept 23	Aflibercept+CLS-TA 23
Feltgen, 2018 (45)	NCT01580020	92	BRVO	Dexamethasone 62	Ranibizumab 113	NA
Gado, 2014 (46)	NA	60	CRVO	Dexamethasone 30	Ranibizumab 30	NA
Ghader, 2017 (47)	TCTR20170612005	90	CRVO	Triamcinolone 30	Bevacizumab 30	Triamcinolone+Bevacizumab 30
Hattenbach, 2018 (48)	NCT01396057	244	BRVO	Dexamethasone 118	Ranibizumab 126	NA
Hoerauf, 2016 (49)	NCT01396083	243	CRVO	Dexamethason 119	Ranibizumab 124	NA
Kumar, 2019 (50)	NA	30	BRVO	Dexamethason 15	Ranibizumab 15	NA
Limon, 2022 (51)	NA	67	BRVO	NA	Bevacizumab 35	Bevacizumab+Dexamethason 32
Lucatto, 2017 (52)	NA	35	CRVO	Triamcinolone 11	Bevacizumab 14	NA
Maturi, 2014, (20)	NA	30	RVO	NA	Bevacizumab 15	Bevacizumab+Dexamethason 15
Meng, 2024 (53)	NA	292	BRVO	Dexamethason 98	Ranibizumab 96	Ranibizumab+Dexamethason 98
Moon, 2016 (54)	NCT01614509	45	BRVO	NA	Bevacizumab 23	Bevacizumab+Triamcinolone 18
Osman, 2010 (55)	NA	52	BRVO	Triamcinolone 17	Bevacizumab 14	Bevacizumab+Triamcinolone 21
Rahman, 2025 (56)	NA	100	RVO	Dexamethasone 50	Ranibizumab or Aflibercept 50	NA
Ramezani, 2012 (57)	NCT01044329	86	BRVO	Dexamethasone 43	Bevacizumab 43	NA
Ramezani, 2014 (58)	NCT01178697	86	CRVO	Triamcinolone 43	Bevacizumab 43	NA
Tomoaki, 2013 (59)	UMIN000001546	43	BRVO	Triamcinolone 21	Ranibizumab 22	NA
Wang, 2011 (60)	NA	75	CRVO	NA	Bevacizumab 36	Bevacizumab+Triamcinolone 39
Xiao, 2011 (61)	NA	31	CRVO	Triamcinolone 16	Bevacizumab 16	NA
Zhao, 2020 (9001)	ChiCTR1900028003	53	BRVO	Triamcinolone 17	Conbercept 36	NA

RVO, retinal vein occlusion; CRVO, central retinal vein occlusion; BRVO, branch retinal vein occlusion; CLS-TA, Triamcinolone Acetonide; Anti-VEGF, Anti-Vascular Endothelial Growth Factor, Combination therapy: Steroids+Anti-VEGF.

### 3.3 Risk of bias and evidence certainty

**Figure 2:**
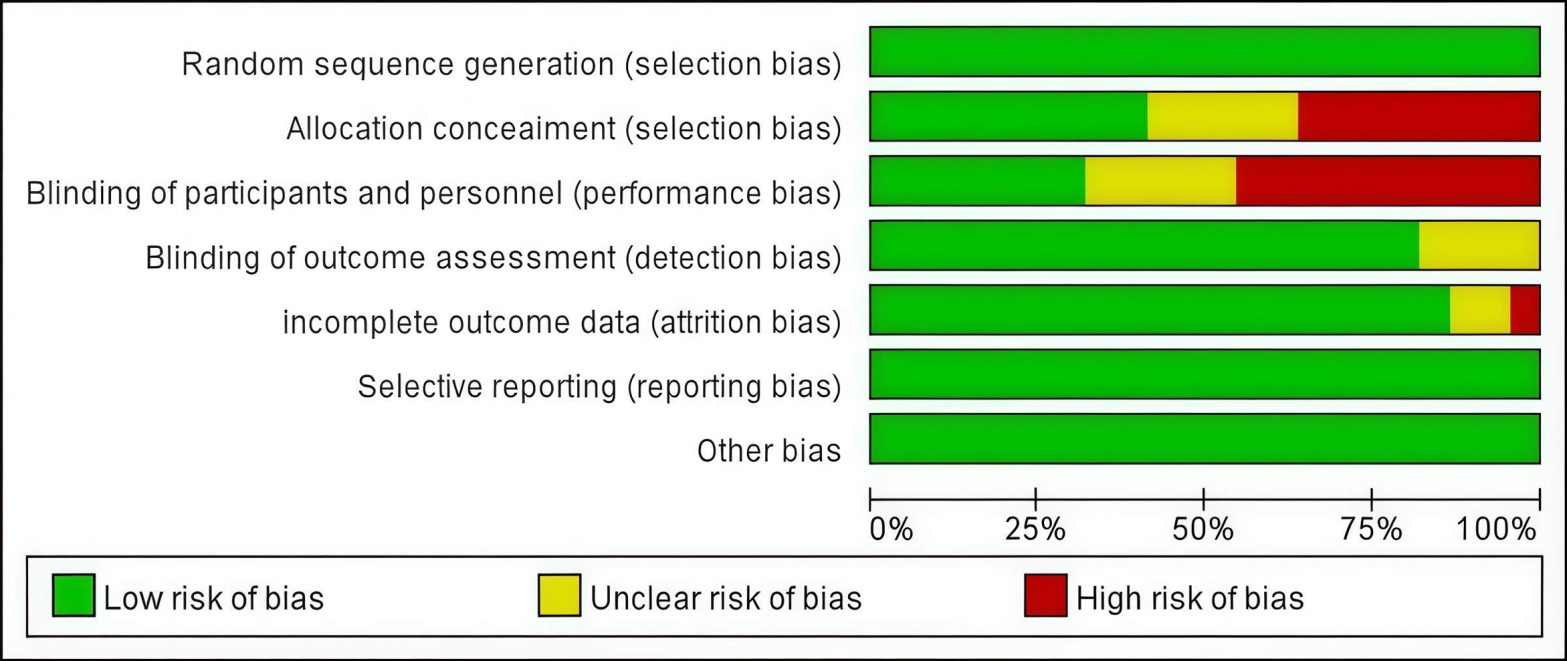
Risk of bias graph.

The risk of bias was assessed using the Cochrane risk-of-bias tool for randomized trials. Figure 2 presents a concise overview of the risk of bias assessment results.

**Figure 3:**
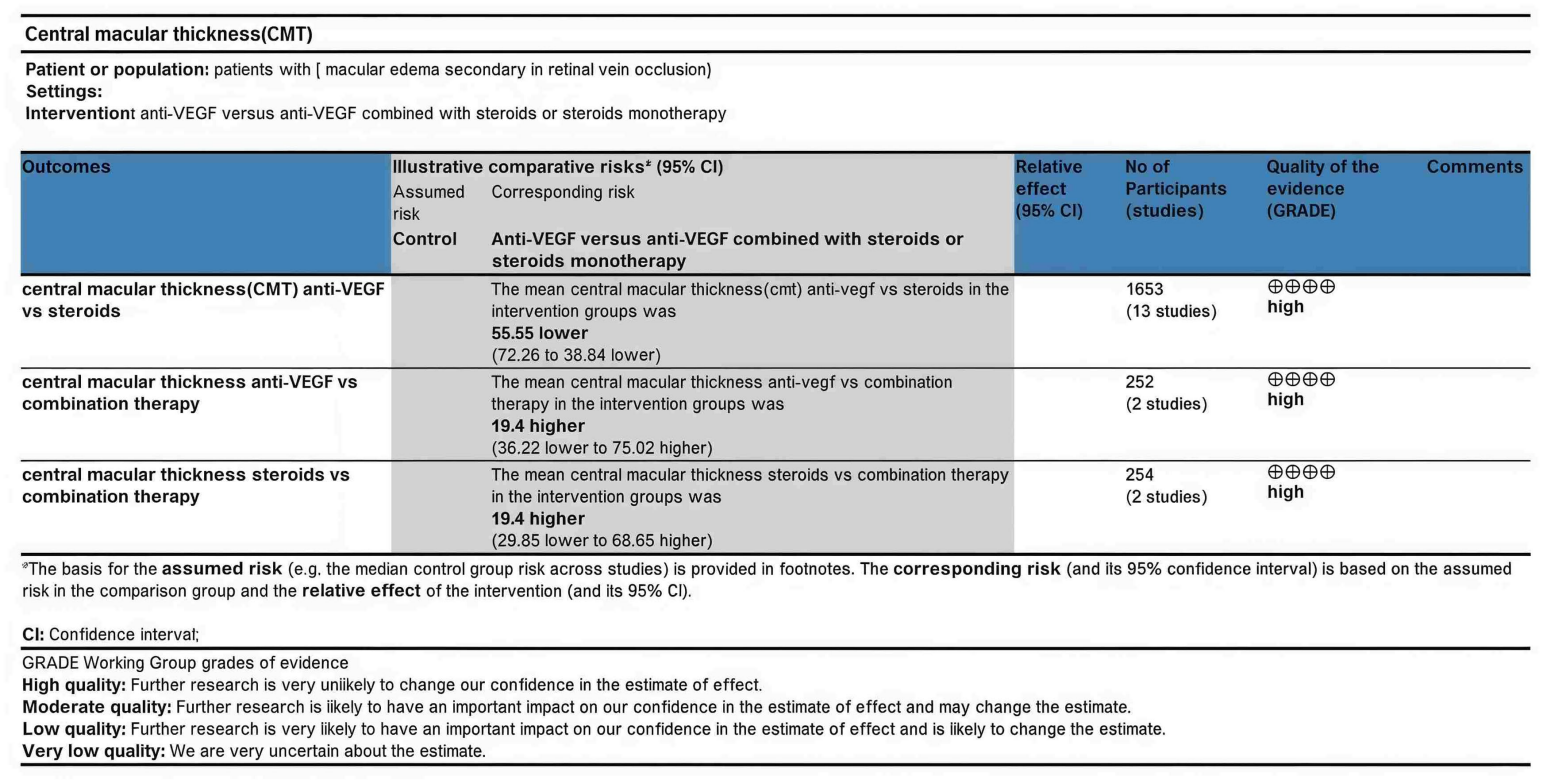
Quality assessment of the evidence using GRADEprofiler.

The certainty of evidence for the systematic review was evaluated using GRADEpro GDT. The overall quality of evidence for the comparisons of anti-VEGF monotherapy versus combination therapy with steroids or steroid monotherapy in terms of changes in central macular thickness (CMT) was rated as high (Figure 3).

### 3.4 Best-corrected visual acuity

**Figure 4:**
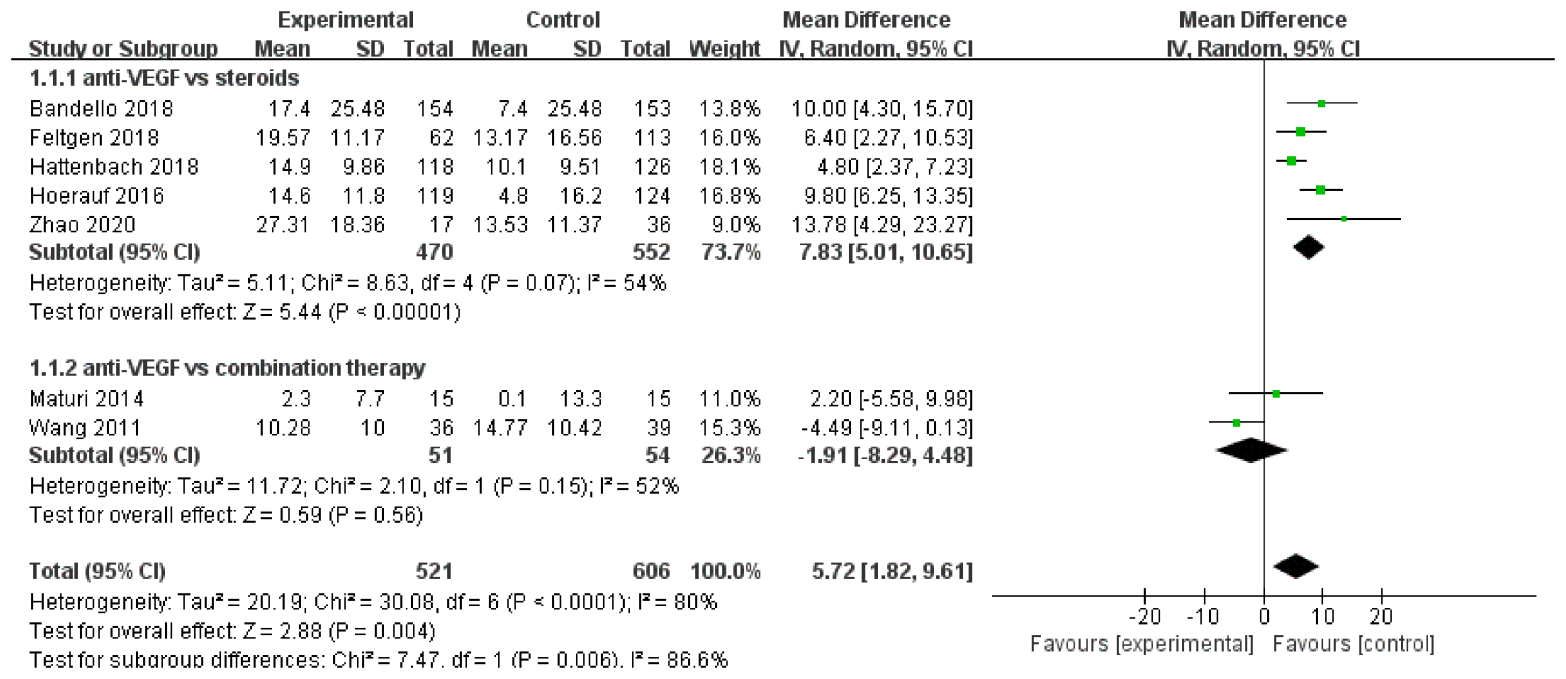
Forest plot of the meta-analysis for the Early Treatment Diabetic Retinopathy Study (ETDRS) outcomes.

A total of seven trials used the Early Treatment Diabetic Retinopathy Study (ETDRS) letters to assess changes in best-corrected visual acuity. The pooled results (mean difference, 5.72, 95% CI, 1.82 to 9.61, *p* = 0.004) indicated that anti-VEGF monotherapy was more effective compared to the other two treatments (Figure 4). In the subgroup analysis, compared to corticosteroids, anti-VEGF monotherapy (mean difference, 7.83, 95% CI, 5.01 to 10.65, *p* < 0.00001) showed a more significant improvement in best-corrected visual acuity. Anti-VEGF monotherapy showed a similar therapeutic effect to combination therapy (mean difference, −1.91, 95% CI, −8.29 to 4.48, *p* = 0.56) (Figure 4).

**Figure 5:**
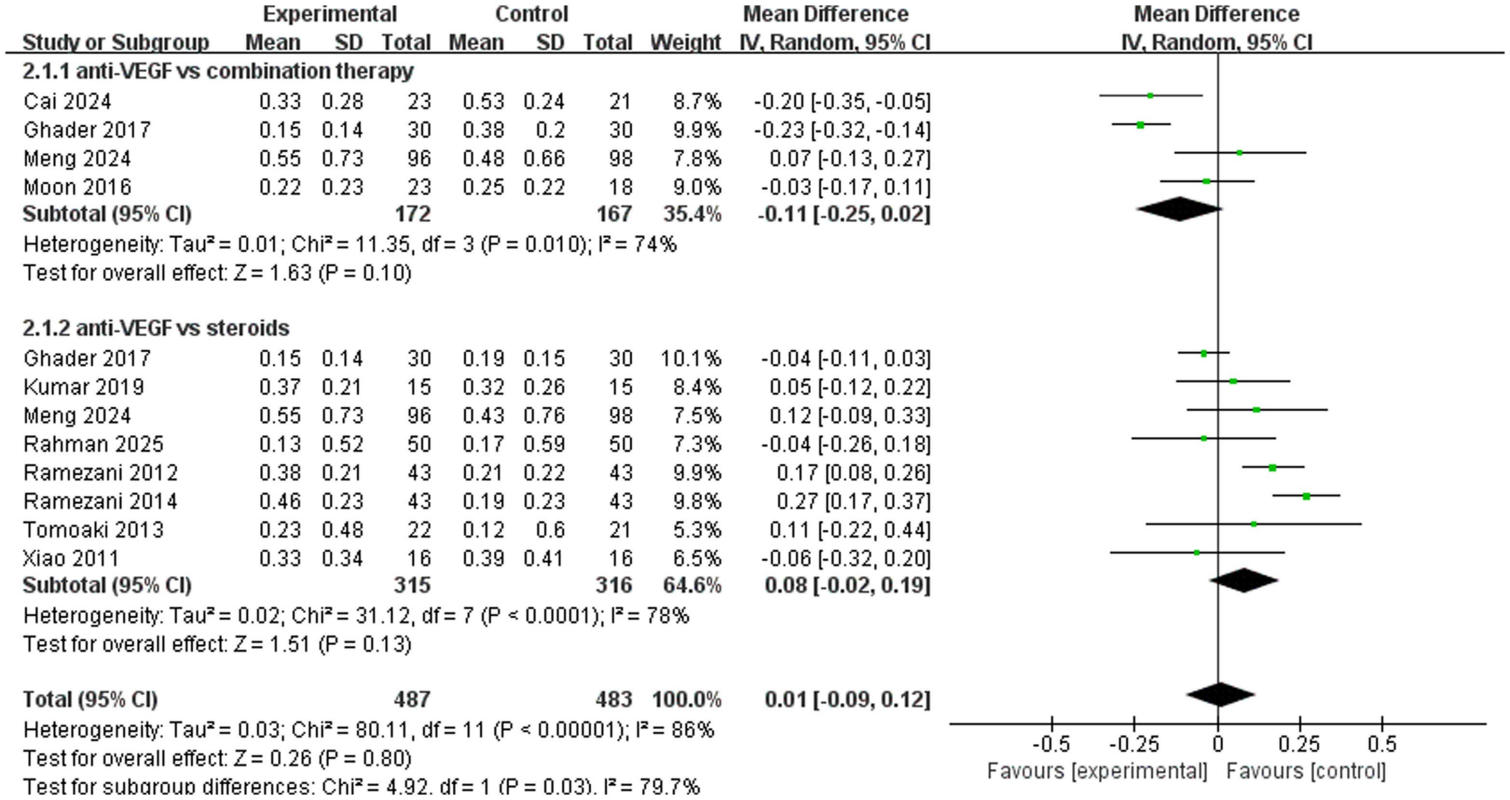
Forest plot of the meta-analysis for logMAR.

A total of 10 trials used the standard logarithm of the minimum angle of resolution (logMAR) VA chart to assess best-corrected visual acuity. The pooled results (mean difference, 0.01, 95% CI, −0.09 to 0.12, *p* = 0.80) indicated that anti-VEGF monotherapy had no significant advantage over combination therapy or steroid monotherapy. In the subgroup analysis, four trials (mean difference, −0.11, 95% CI, −0.25 to 0.02, *p* = 0.10) compared anti-VEGF monotherapy with combination therapy and found similar therapeutic effects. A total of eight trials (mean difference, 0.08, 95% CI, −0.02 to 0.19, *p* = 0.13) compared anti-VEGF monotherapy with steroid monotherapy, and their therapeutic efficacy was comparable (Figure 5).

### 3.5 Central macular thickness

**Figure 6:**
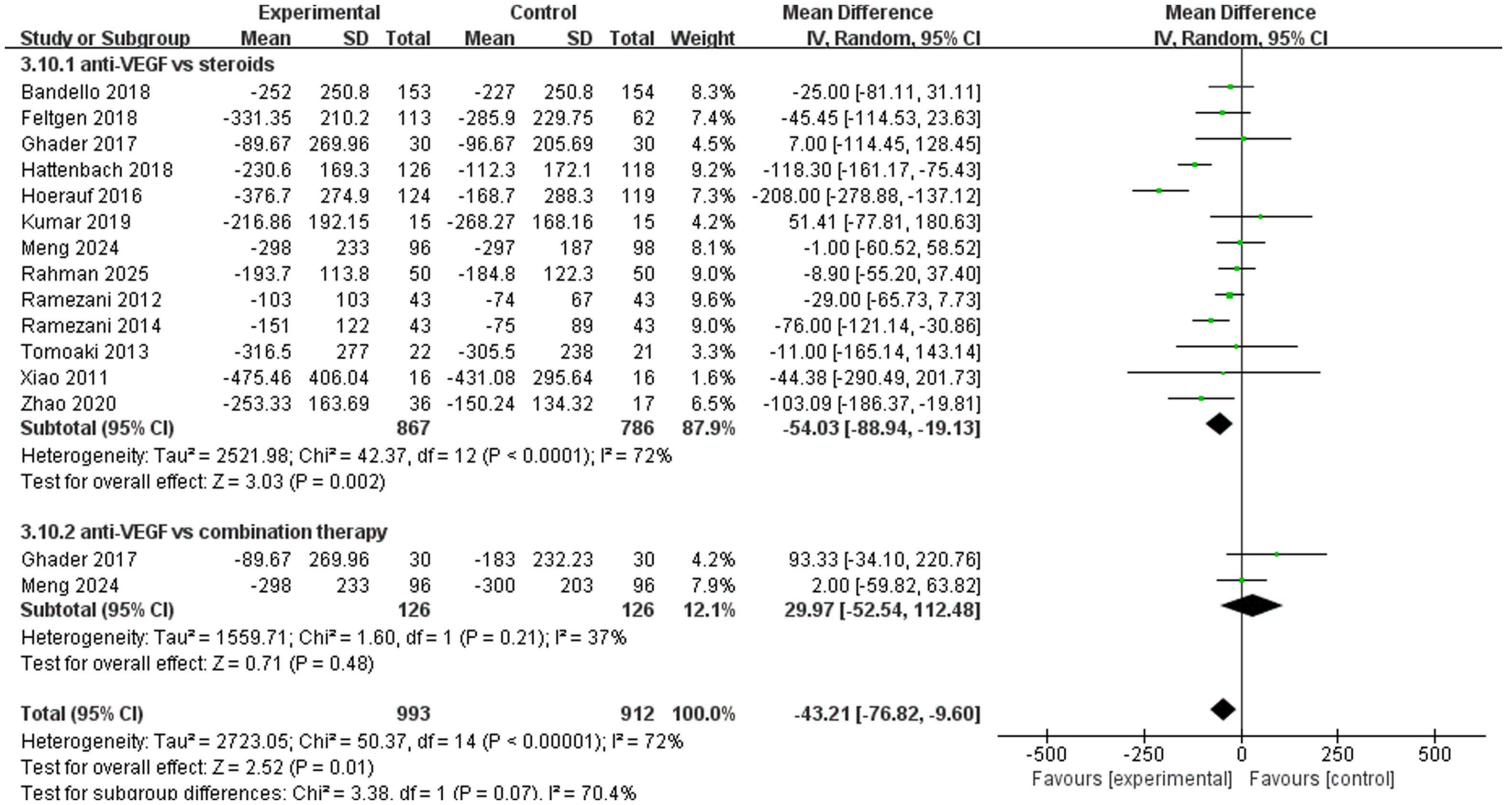
Forest plot of the meta-analysis for CMT.

A total of 13 trials analyzed improvements in central macular thickness. The pooled results (mean difference, −43.21, 95% CI, −76.82 to −9.60, *p* = 0.01) indicated that anti-VEGF monotherapy was more effective compared to the other two treatments (Figure 6). In the subgroup analysis, two trials (mean difference, 29.97, 95% CI, −52.54 to 112.48, *p* = 0.48) compared anti-VEGF monotherapy with combination therapy and found similar therapeutic effects. A total of 13 trials (mean difference, −54.03, 95% CI, −88.94 to −19.13, *p* = 0.002) compared anti-VEGF monotherapy with steroid monotherapy, and anti-VEGF monotherapy demonstrated superior efficacy (Figure 6).

### 3.6 Number of injections

**Figure 7:**
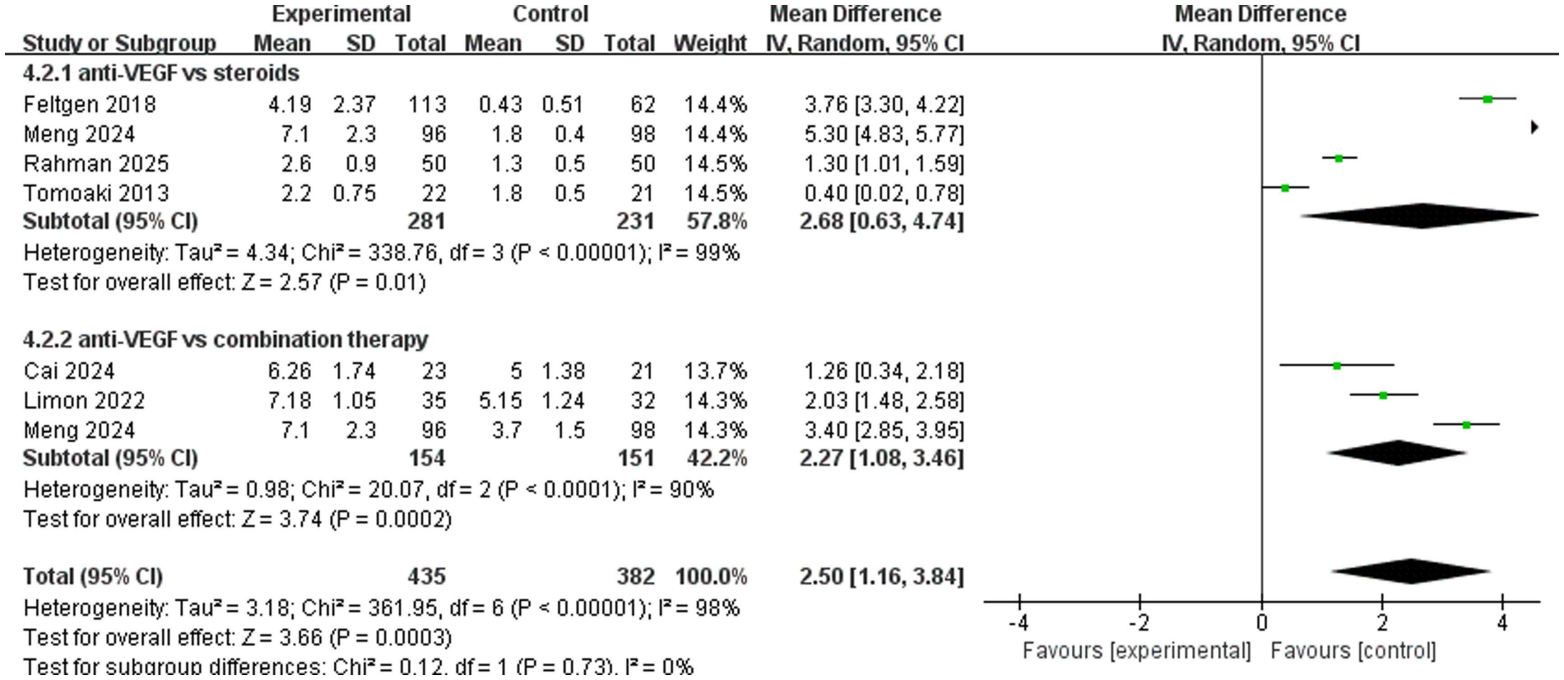
Forest plot of the meta-analysis for the number of injections.

A total of six trials analyzed the number of injections. The pooled results (mean difference, 2.50, 95% CI, 1.16 to 3.84, *p* = 0.0003) indicated that anti-VEGF monotherapy required more injections compared to the other two treatments (Figure 7). In the subgroup analysis, four trials (mean difference, 2.68, 95% CI, 0.63 to 4.74, *p* = 0.01) compared anti-VEGF monotherapy with steroid monotherapy and showed that anti-VEGF monotherapy required more injections. A total of three trials (mean difference, 2.27, 95% CI, 1.08 to 3.46, *p* = 0.0002) compared anti-VEGF monotherapy with combination therapy, with combination therapy showing the advantage of requiring fewer injections (Figure 7).

### 3.7 Incidence of adverse events

A total of eight adverse events were included in the analysis: cataract, conjunctival hemorrhage, eye pain, intraocular pressure (IOP), increased lacrimation, macular edema, ocular hypertension, and reduced visual acuity. Compared to steroid monotherapy, anti-VEGF monotherapy can reduce the incidence of cataract, elevated intraocular pressure, ocular hypertension, and reduced visual acuity. In addition, compared to combination therapy, anti-VEGF monotherapy can reduce the occurrence of IOP. The detailed results are presented in the Supplementary material.

### 3.8 Sensitivity analyses

**Figure 8:**
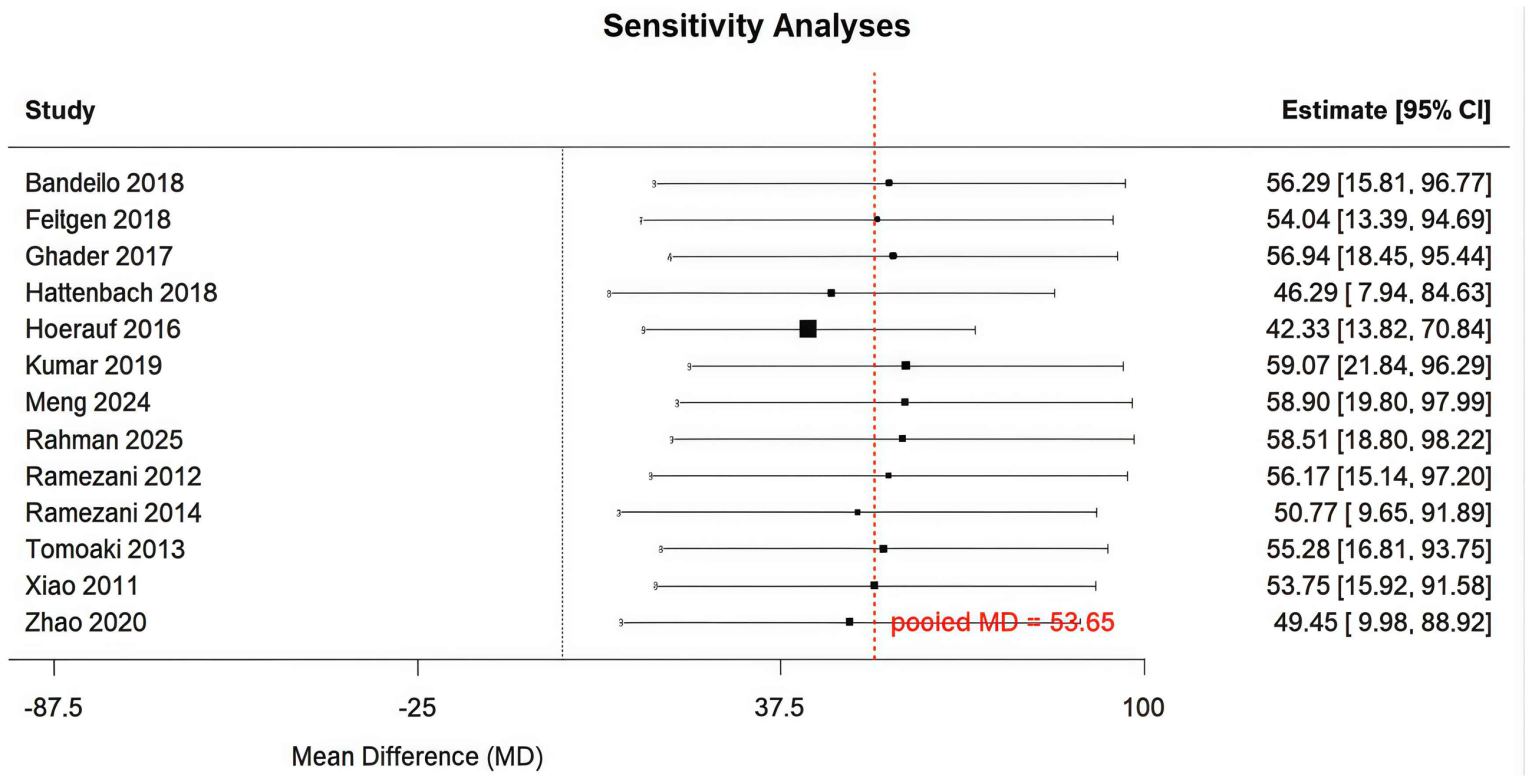
Sensitivity analysis of changes in central macular thickness.

A sensitivity analysis was conducted on changes in central macular thickness. The pooled results remained unchanged following the sequential exclusion of each trial. The sensitivity analysis indicated that the results were stable. Further details are provided in Figure 8.

### 3.9 Publication Bias

**Figure 9:**
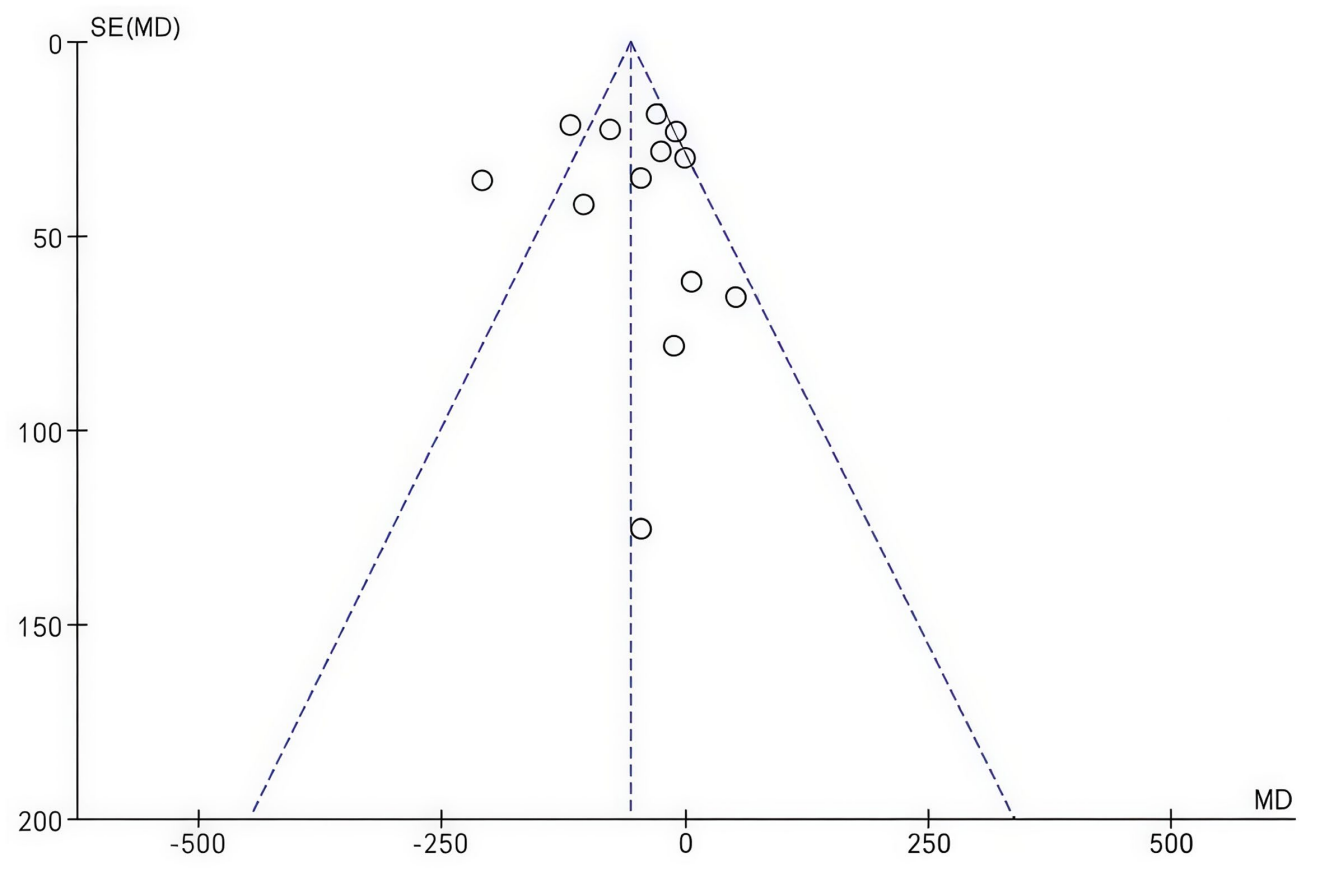
Funnel plot of changes in central macular thickness.

Publication bias was assessed using a funnel plot for changes in central macular thickness (Figure 9). The symmetrical appearance of the funnel plot indicated no significant evidence of publication bias (Begg’s test, *p* = 0.5025).

## 4 Discussion

This meta-analysis included 22 RCTs to evaluate the efficacy of intravitreal anti-vascular endothelial growth factor (anti-VEGF) drugs compared to corticosteroids and combination therapy for the treatment of macular edema (ME) secondary to retinal vein occlusion (RVO). Among these 22 trials, three compared the efficacy of all three treatment modalities, six specifically assessed anti-VEGF monotherapy versus combination therapy, and 13 evaluated corticosteroid monotherapy versus anti-VEGF monotherapy. Regarding anatomical outcomes, anti-VEGF agents demonstrated superiority over corticosteroids in reducing central macular thickness (CMT). This finding contrasts sharply with those of Tang, Xiaodong, and Qiu et al. (26, 32, 33), who reported that corticosteroids were more effective in reducing CMT.

In terms of visual outcomes, for logarithm of the minimum angle of resolution (logMAR) visual acuity (VA), the efficacy of anti-VEGF monotherapy was not different from that of corticosteroid therapy or combination therapy. Anti-VEGF monotherapy also provided greater benefits for VA when measured using the Early Treatment Diabetic Retinopathy Study (ETDRS) letter scale. Notably, our findings regarding improvements in best-corrected visual acuity (BCVA)—a composite measure encompassing both logMAR VA and ETDRS letters—are not entirely consistent with those reported by Tang et al. (26).

Furthermore, our results indicated that anti-VEGF agents were effective in preventing intraocular pressure (IOP) elevation compared to the other two treatment regimens. This finding is consistent with safety data from multiple studies (26, 29, 32, 33), which confirmed that intravitreal anti-VEGF agents have a more favorable safety profile than corticosteroids. With respect to combination therapy, Namvar et al. and Zhang et al. (27, 28) previously demonstrated that anti-VEGF plus corticosteroid combination therapy outperforms monotherapy (whether corticosteroid monotherapy or intravitreal anti-VEGF monotherapy). Regrettably, only four trials in our meta-analysis compared the effects of combination therapy on BCVA improvement. Among these, anti-VEGF monotherapy was found to be more effective in enhancing logMAR VA, a key component of BCVA. For other outcome measures, the interpretability of our results may be limited due to the insufficient available data.

Anti-vascular endothelial growth factor (anti-VEGF) agents significantly reduce central macular thickness (CMT) and improve visual acuity in patients with RVO-associated macular edema (ME) through three core mechanisms: The inhibition of vascular leakage, the blockade of abnormal angiogenesis, and the preservation of photoreceptor structural integrity. This conclusion is supported by numerous research studies and clinical trials. First, the inhibition of VEGF-mediated elevated vascular permeability serves to directly alleviate macular edema. One of the core biological functions of VEGF is its role as a “vascular permeability factor.” By activating vascular endothelial growth factor receptor 2 (VEGFR-2) on endothelial cells, VEGF induces the relaxation of interendothelial tight junctions and increases calcium influx. This disrupts the blood–retinal barrier (BRB) and leads to the extravasation of intravascular fluid into the retinal neuroepithelium, contributing to the development of macular edema (34). In patients with RVO (including branch retinal vein occlusion [BRVO] and central retinal vein occlusion [CRVO]), VEGF levels in the aqueous and vitreous humor are significantly elevated. Moreover, VEGF concentration is positively correlated with the severity of macular edema, as assessed by OCT-measured CMT (35). Specifically, the median aqueous VEGF level in BRVO patients reaches 351 pg./mL (compared to 119 pg./mL in the control group), and VEGF levels exhibit significant correlations with both the area of retinal non-perfusion and CMT. Anti-VEGF agents (e.g., ranibizumab, aflibercept) can directly bind to all isoforms of VEGF-A (including VEGF₁₂₁ and VEGF₁₆₅), preventing their interaction with VEGFR-1/VEGFR-2 and blocking the downstream PI3K-Akt signaling pathway, which stabilizes interendothelial tight junctions and reduces vascular leakage (34, 36). Clinical data show that 6 months of ranibizumab treatment results in a mean CMT reduction of 337–345 μm in BRVO patients and 434–452 μm in CRVO patients, which is significantly greater than that observed in the sham group (37, 38). Second, Blockade of Ischemia-Induced Abnormal Angiogenesis to Improve Retinal Microcirculation. RVO impairs retinal venous reflux, leading to local ischemia and hypoxia. Hypoxia upregulates VEGF expression via hypoxia-inducible factor-1 (HIF-1), thereby promoting abnormal angiogenesis (39). The newly formed blood vessels are structurally disorganized and highly permeable, which further exacerbates edema and hemorrhage while compressing normal retinal tissue and impairing visual conduction. Using optical coherence tomography angiography (OCTA), a study (40) confirmed that anti-VEGF therapy can significantly improve retinal microcirculation after RVO, reduce the formation of abnormal vascular networks, and decrease the positive rate of vascular leakage markers (e.g., fluorescein leakage). Clinical studies have demonstrated that after ranibizumab treatment, the leakage rate associated with abnormal blood vessels in CRVO patients decreases from 7 to 0.8–1.5%. Similarly, treatment with aflibercept (VEGF Trap-Eye) reduces the incidence of neovascularization in CRVO patients from 4.4 to 2.9% (36, 38), which indirectly reduces edema recurrence and helps maintain CMT reduction. Third, Preservation of Photoreceptor Structural Integrity to Restore Visual Function. The persistence of macular edema directly compresses and damages photoreceptor microstructures, such as the inner segment/outer segment (IS/OS) junction and external limiting membrane (ELM), leading to impairment of visual signal transmission (41). By rapidly alleviating edema, anti-VEGF agents provide a stable microenvironment for photoreceptors, preventing irreversible damage. Research has confirmed that RVO-associated edema results in thinning of the photoreceptor layer (mean thickness: 71.1 ± 26.8 μm) and disruption or loss of the IS/OS junction (41). Moreover, visual acuity is directly correlated with photoreceptor layer thickness and IS/OS integrity (41). Clinical trials show that after 6 months of ranibizumab treatment, 61.1% of BRVO patients and 47.7% of CRVO patients achieve a best-corrected visual acuity (BCVA) improvement of ≥15 ETDRS letters. This visual improvement is positively correlated with CMT reduction and IS/OS junction recovery (37, 38). Similarly, aflibercept treatment results in a BCVA improvement of ≥15 ETDRS letters in 60.2% of CRVO patients, with a mean CMT reduction of 448.6 μm (36). 4. Blockade of VEGFR Downstream Signaling Pathways to Inhibit Abnormal Endothelial Cell Proliferation. VEGF binding to VEGFR-2 activates signaling pathways such as Raf–Mek–Erk and PI3K-Akt, which promote the proliferation and migration of vascular endothelial cells. This leads to excessive retinal vascular proliferation and a sustained increase in vascular wall permeability (34). Anti-VEGF agents completely block this signal transduction: ranibizumab, a humanized monoclonal antibody fragment, exhibits an affinity for VEGF that is more than 100 times higher than that of natural receptors (38); aflibercept, a VEGFR-1/VEGFR-2 fusion protein, can simultaneously block VEGF-A and placental growth factor (PLGF), exerting a dual inhibitory effect on vascular proliferation signals (36). Ultimately, this reduces the compression of the macular region by abnormal blood vessels and mitigates edema progression. Anti-VEGF agents achieve dual benefits of “CMT reduction” and “visual acuity improvement” through the synergistic effects of “symptomatic treatment (direct blockade of vascular leakage), etiological treatment (inhibition of abnormal angiogenesis), and functional recovery (preservation of photoreceptor structure).”

The current study has several strengths. First, we included 22 randomized controlled trials in the meta-analysis, rather than other types of studies, as RCTs are considered high-quality evidence for evaluating treatment efficacy. Second, this meta-analysis is an updated version and therefore provides more representative and current evidence. We implemented a systematic and comprehensive database search strategy without date restrictions across major online databases (PubMed, Embase, the Cochrane Library, and Web of Science) to avoid the influence of publication bias on the pooled results and improve the reproducibility of the results. Third, the quality of evidence for all included results was evaluated using the Cochrane Collaboration tools. Fourth, this study explored the efficacy and safety of three treatment regimens for macular edema secondary to retinal vein occlusion, providing a comprehensive data analysis.

Our study has several limitations. First, although 22 RCTs were included, the number of trials available for certain analyses remains relatively small, and the data are limited. For instance, regarding CMT, 13 trials compared the effects of anti-vascular endothelial growth factor therapy with corticosteroids, but only two trials compared the combination treatment with either corticosteroids or anti-VEGF therapy. Therefore, the limited data made it impossible for this meta-analysis to assess the advantages and disadvantages of combination therapy. In addition, regarding BCVA and adverse events, the small number of included trials made it impossible to clearly determine the advantages and disadvantages of the three treatment modalities. Second, the follow-up periods for each trial were different, making it impossible to conduct monthly subgroup analyses to evaluate changes in CTM and BCVA each month after receiving the three different treatment modalities. Thirdly, subgroup analysis of specific drugs cannot be performed using either anti-vascular endothelial factor or cortisol to explore the therapeutic effects of different agents. Fourth, most trials did not record adverse events in detail, which affected our assessment of the incidence of adverse events. Fifth, differences in drug types, injection doses, and injection frequencies inevitably introduced heterogeneity across studies. Sixth, the evaluation of the efficacy of intravitreal anti-VEGF and corticosteroid therapies in BRVO and CRVO remains limited.

## 5 Conclusion

This meta-analysis indicates that anti-VEGF monotherapy is more effective than the other two treatment modalities in reducing CMT in patients with retinal vein occlusion. Regarding BCVA, anti-VEGF monotherapy is more effective than the other two treatments when assessed using the ETDRS score. In addition, compared to the other two treatment modalities, anti-VEGF monotherapy is more effective in lowering postoperative intraocular pressure levels and reducing the incidence of cataract, ocular hypertension, and reduced visual acuity. Given the limitations of this study, it is crucial to conduct multi-center randomized controlled trials to assess the safety and efficacy of the three treatment modalities and to increase the sample size to validate our results.

## Data Availability

The datasets presented in this study can be found in online repositories. The names of the repository/repositories and accession number(s) can be found in the article/Supplementary material.

## References

[1] Maggio E Mete M Maraone G Attanasio M Guerriero M Pertile G. Intravitreal injections for macular edema secondary to retinal vein occlusion: long-term functional and anatomic outcomes. J Ophthalmol. (2020) 2020:1–8. doi: 10.1155/2020/7817542, 32104597 PMC7040414

[2] Song P Xu Y Zha M Zhang Y Rudan I. Global epidemiology of retinal vein occlusion: a systematic review and Meta-analysis of prevalence, incidence, and risk factors. J Glob Health. (2019) 9:010427. doi: 10.7189/jogh.09.010427, 31131101 PMC6513508

[3] Kalva P Akram R Zuberi HZ Kooner KS. Prevalence and risk factors of retinal vein occlusion in the United States: the National Health and nutrition examination survey, 2005 to 2008. Proceedings. (2023) 36:335–40. doi: 10.1080/08998280.2023.2173938, 37091777 PMC10120443

[4] Flaxel CJ Adelman RA Bailey ST Fawzi A Lim JI Vemulakonda GA . Retinal vein occlusions preferred practice pattern®. Ophthalmology. (2020) 127:P288–p320. doi: 10.1016/j.ophtha.2019.09.029, 31757503

[5] Spooner K Fraser-Bell S Hong T Chang A. Effects of switching to Aflibercept in treatment resistant macular edema secondary to retinal vein occlusion. Asia Pac J Ophthalmol. (2020) 9:48–53. doi: 10.1097/01.apo.0000617924.11529.88, 31990746 PMC7004466

[6] Daruich A Matet A Moulin A Kowalczuk L Nicolas M Sellam A . Mechanisms of macular edema: beyond the surface. Prog Retin Eye Res. (2018) 63:20–68. doi: 10.1016/j.preteyeres.2017.10.006, 29126927

[7] Romano F Lamanna F Gabrielle PH Teo KYC Battaglia Parodi M Iacono P . Update on retinal vein occlusion. Asia Pac J Ophthalmol. (2023) 12:196–210. doi: 10.1097/apo.0000000000000598, 36912792

[8] Branch Vein Occlusion Study Group. Argon laser photocoagulation for macular edema in branch vein occlusion. The branch vein occlusion study group. Am J Ophthalmol. (1984) 98:271–82. doi: 10.1016/0002-9394(84)90316-7 6383055

[9] Haller JA Bandello F Belfort R Blumenkranz MS Gillies M Heier J . Randomized, sham-controlled trial of dexamethasone intravitreal implant in patients with macular edema due to retinal vein occlusion. Ophthalmology. (2010) 117:1134–46. doi: 10.1016/j.ophtha.2010.03.032 20417567

[10] The Central Vein Occlusion Study Group. Evaluation of grid pattern photocoagulation for macular edema in central vein occlusion. Ophthalmology. (1995) 102:1425–33. doi: 10.1016/s0161-6420(95)30849-4 9097788

[11] Ip MS Scott IU VanVeldhuisen PC Oden NL Blodi BA Fisher M . A randomized trial comparing the efficacy and safety of intravitreal triamcinolone with observation to treat vision loss associated with macular edema secondary to central retinal vein occlusion: the standard care vs corticosteroid for retinal vein occlusion (score) study report 5. Arch Ophthalmol. (2009) 127:1101–14. doi: 10.1001/archophthalmol.2009.234 19752419 PMC2872173

[12] Tah V Orlans HO Hyer J Casswell E Din N Sri Shanmuganathan V . Anti-VEGF therapy and the retina: an update. J Ophthalmol. (2015) 2015:627674. doi: 10.1155/2015/627674, 26417453 PMC4568374

[13] Rhoades W Dickson D Nguyen QD Do DV. Management of Macular Edema due to central retinal vein occlusion - the role of Aflibercept. Taiwan J Ophthalmol. (2017) 7:70–6. doi: 10.4103/tjo.tjo_9_17, 29018760 PMC5602151

[14] Schmidt-Erfurth U Garcia-Arumi J Gerendas BS Midena E Sivaprasad S Tadayoni R . Guidelines for the Management of Retinal Vein Occlusion by the European Society of Retina Specialists (Euretina). Ophthalmologica. (2019) 242:123–62. doi: 10.1159/000502041, 31412332

[15] Ehlers JP Kim SJ Yeh S Thorne JE Mruthyunjaya P Schoenberger SD . Therapies for macular edema associated with branch retinal vein occlusion: a report by the American Academy of ophthalmology. Ophthalmology. (2017) 124:1412–23. doi: 10.1016/j.ophtha.2017.03.060, 28551163

[16] Garweg JG Wenzel A. Diabetic maculopathy and retinopathy. Functional and Sociomedical significance. Ophthalmologe. (2010) 107:628–35. doi: 10.1007/s00347-010-2176-x 20533047

[17] Fogli S Mogavero S Egan CG Del Re M Danesi R. Pathophysiology and pharmacological targets of Vegf in diabetic macular edema. Pharmacol Res. (2016) 103:149–57. doi: 10.1016/j.phrs.2015.11.003, 26607863

[18] Nicholson L Talks SJ Amoaku W Talks K Sivaprasad S. Retinal vein occlusion (Rvo) guideline: executive summary. Eye. (2022) 36:909–12. doi: 10.1038/s41433-022-02007-4, 35301458 PMC9046155

[19] Qian T Zhao M Wan Y Li M Xu X. Comparison of the efficacy and safety of drug therapies for macular edema secondary to central retinal vein occlusion. BMJ Open. (2018) 8:e022700. doi: 10.1136/bmjopen-2018-022700, 30593547 PMC6318534

[20] Maturi RK Chen V Raghinaru D Bleau L Stewart MW. A 6-month, subject-masked, randomized controlled study to assess efficacy of dexamethasone as an adjunct to bevacizumab compared with bevacizumab alone in the treatment of patients with macular edema due to central or branch retinal vein occlusion. Clin Ophthalmol. (2014) 8:1057–64. doi: 10.2147/opth.s60159, 24940042 PMC4051812

[21] Campochiaro PA Brown DM Awh CC Lee SY Gray S Saroj N . Sustained benefits from Ranibizumab for macular edema following central retinal vein occlusion: twelve-month outcomes of a phase iii study. Ophthalmology. (2011) 118:2041–9. doi: 10.1016/j.ophtha.2011.02.038, 21715011

[22] Brown DM Heier JS Clark WL Boyer DS Vitti R Berliner AJ . Intravitreal Aflibercept injection for macular edema secondary to central retinal vein occlusion: 1-year results from the phase 3 Copernicus study. Am J Ophthalmol. (2013) 155:429–437.e7. doi: 10.1016/j.ajo.2012.09.026, 23218699

[23] Wang N Hunt A Nguyen V Shah J Fraser-Bell S McAllister I . One-year real-world outcomes of bevacizumab for the treatment of macular Oedema secondary to retinal vein occlusion. Clin Experiment Ophthalmol. (2022) 50:1038–46. doi: 10.1111/ceo.14139, 35869925

[24] Hykin P Prevost AT Vasconcelos JC Murphy C Kelly J Ramu J . Clinical effectiveness of intravitreal therapy with ranibizumab vs aflibercept vs bevacizumab for macular edema secondary to central retinal vein occlusion: a randomized clinical trial. JAMA Ophthalmol. (2019) 137:1256–64. doi: 10.1001/jamaophthalmol.2019.3305, 31465100 PMC6865295

[25] Spooner KL Fraser-Bell S Hong T Wong JG Chang AA. Long-term outcomes of anti-Vegf treatment of retinal vein occlusion. Eye. (2022) 36:1194–201. doi: 10.1038/s41433-021-01620-z, 34117379 PMC9151794

[26] Tang HX Li JJ Yuan Y Ling Y Mei Z Zou H. Comparing the efficacy of dexamethasone implant and anti-Vegf for the treatment of macular edema: a systematic review and Meta-analysis. PLoS One. (2024) 19:e0305573. doi: 10.1371/journal.pone.0305573, 38985778 PMC11236136

[27] Namvar E Yasemi M Nowroozzadeh MH Ahmadieh H. Intravitreal injection of anti-vascular endothelial growth factors combined with corticosteroids for the treatment of macular edema secondary to retinal vein occlusion: a systematic review and Meta-analysis. Semin Ophthalmol. (2024) 39:109–19. doi: 10.1080/08820538.2023.2249527, 37621098

[28] Zhang WY Liu Y Sang AM. Efficacy and effectiveness of anti-Vegf or steroids monotherapy versus combination treatment for macular edema secondary to retinal vein occlusion: a systematic review and Meta-analysis. BMC Ophthalmol. (2022) 22:472. doi: 10.1186/s12886-022-02682-7, 36474156 PMC9727869

[29] Patil NS Hatamnejad A Mihalache A Popovic MM Kertes PJ Muni RH. Anti-vascular endothelial growth factor treatment compared with steroid treatment for retinal vein occlusion: a meta-analysis. Ophthalmologica. (2023) 245:500–15. doi: 10.1159/000527626, 36288721

[30] Cornish EE Zagora SL Spooner K Fraser-Bell S. Management of Macular Oedema due to retinal vein occlusion: an evidence-based systematic review and Meta-analysis. Clin Experiment Ophthalmol. (2023) 51:313–38. doi: 10.1111/ceo.14225, 37060158

[31] Page MJ Moher D Bossuyt PM Boutron I Hoffmann TC Mulrow CD . Prisma 2020 explanation and elaboration: updated guidance and exemplars for reporting systematic reviews. BMJ. (2021) 372:n160. doi: 10.1136/bmj.n160, 33781993 PMC8005925

[32] Xiaodong L Xuejun X. The efficacy and safety of dexamethasone intravitreal implant for diabetic macular edema and macular edema secondary to retinal vein occlusion: a Meta-analysis of randomized controlled trials. J Ophthalmol. (2022) 2022:1–11. doi: 10.1155/2022/4007002, 35982771 PMC9381227

[33] Qiu XY Hu XF Qin YZ Ma JX Liu QP Qin L . Comparison of intravitreal aflibercept and dexamethasone implant in the treatment of macular edema associated with diabetic retinopathy or retinal vein occlusion: a meta-analysis and systematic review. Int J Ophthalmol. (2022) 15:1511–9. doi: 10.18240/ijo.2022.09.15, 36124196 PMC9453398

[34] Ferrara N Gerber HP LeCouter J. The biology of VEGF and its receptors. Nat Med. (2003) 9:669–76. doi: 10.1038/nm0603-669, 12778165

[35] Noma H Funatsu H Yamasaki M Tsukamoto H Mimura T Sone T . Pathogenesis of macular edema with branch retinal vein occlusion and intraocular levels of vascular endothelial growth factor and Interleukin-6. Am J Ophthalmol. (2005) 140:256–61. doi: 10.1016/j.ajo.2005.03.003, 16086947

[36] Holz FG Roider J Ogura Y Korobelnik JF Simader C Groetzbach G . Vegf trap-eye for macular Oedema secondary to central retinal vein occlusion: 6-month results of the phase iii Galileo study. Br J Ophthalmol. (2013) 97:278–84. doi: 10.1136/bjophthalmol-2012-301504, 23298885

[37] Campochiaro PA Heier JS Feiner L Gray S Saroj N Rundle AC . Ranibizumab for macular edema following branch retinal vein occlusion: six-month primary end point results of a phase iii study. Ophthalmology. (2010) 117:1102–12. doi: 10.1016/j.ophtha.2010.02.021 20398941

[38] Brown DM Campochiaro PA Singh RP Li Z Gray S Saroj N . Ranibizumab for macular edema following central retinal vein occlusion: six-month primary end point results of a phase iii study. Ophthalmology. (2010) 117:1124–33. doi: 10.1016/j.ophtha.2010.02.022 20381871

[39] Campochiaro PA. Molecular pathogenesis of retinal and choroidal vascular diseases. Prog Retin Eye Res. (2015) 49:67–81. doi: 10.1016/j.preteyeres.2015.06.002, 26113211 PMC4651818

[40] Coscas GJ Lupidi M Coscas F Cagini C Souied EH. Optical coherence tomography angiography versus traditional multimodal imaging in assessing the activity of exudative age-related macular degeneration: a new diagnostic challenge. Retina. (2015) 35:2219–28. doi: 10.1097/iae.0000000000000766, 26398697

[41] Ota M Tsujikawa A Murakami T Yamaike N Sakamoto A Kotera Y . Foveal photoreceptor layer in eyes with persistent cystoid macular edema associated with branch retinal vein occlusion. Am J Ophthalmol. (2008) 145:e1:273–80. doi: 10.1016/j.ajo.2007.09.019, 18045566

[42] Bandello F Augustin A Tufail A Leaback R. A 12-month, multicenter, parallel group comparison of dexamethasone intravitreal implant versus ranibizumab in branch retinal vein occlusion. Eur J Ophthalmol. (2018) 28:697–705. doi: 10.1177/1120672117750058 29631435 PMC6210573

[43] Cai X Zhao J Dang Y. Combination Therapy with Anti-VEGF and Intravitreal Dexamethasone Implant for Macular Edema Secondary to Retinal Vein Occlusion. Curr Eye Res. (2024) 49:872–878. doi: 10.1080/02713683.2024.2343055 38639040

[44] Campochiaro PA Wykoff CC Brown DM Boyer DS Barakat M Taraborelli D . Suprachoroidal Triamcinolone Acetonide for Retinal Vein Occlusion: Results of the Tanzanite Study. Ophthalmol Retina. (2018) 2:320–28. doi: 10.1016/j.oret.2017.07.013 31047241

[45] Feltgen N Hattenbach LO Bertelmann T Callizo J Rehak M Wolf A . Comparison of ranibizumab versus dexamethasone for macular oedema following retinal vein occlusion: 1-year results of the COMRADE extension study. Acta Ophthalmol. (2018) 96:e933-e941. doi: 10.1111/aos.13770 29855153

[46] Gado AS Macky TA. Dexamethasone intravitreous implant versus bevacizumab for central retinal vein occlusion-related macular oedema: a prospective randomized comparison. Clin Exp Ophthalmol. (2014) 42:650–5. doi: 10.1111/ceo.1231 24612095

[47] Motarjemizadeh G Rajabzadeh M Aidenloo NS Valizadeh R. Comparison of treatment response to intravitreal injection of triamcinolone, bevacizumab and combined form in patients with central retinal vein occlusion: A randomized clinical trial. Electron Physician. (2017). 9:5068–5074. doi: 10.19082/5068 28979743 PMC5614293

[48] Hattenbach LO Feltgen N Bertelmann T Schmitz-Valckenberg S Berk H Lang GE . Head-to-head comparison of ranibizumab PRN versus single-dose dexamethasone for branch retinal vein occlusion (COMRADE-B). Acta Ophthalmol. (2018) 96:e10-e18. doi: 10.1111/aos.13381 28251811

[49] Hoerauf H Feltgen N Weiss C Paulus EM Schmitz-Valckenberg S Pielen A . Clinical Efficacy and Safety of Ranibizumab Versus Dexamethasone for Central Retinal Vein Occlusion (COMRADE C): A European Label Study. Am J Ophthalmol. (2016) 169:258-267. doi: 10.1016/j.ajo.2016.04.020 27163237

[50] Kumar P Sharma YR Chandra P Azad R Meshram GG. Comparison of the Safety and Efficacy of Intravitreal Ranibizumab with or without Laser Photocoagulation Versus Dexamethasone Intravitreal Implant with or without Laser Photocoagulation for Macular Edema Secondary to Branch Retinal Vein Occlusion. Folia Med (Plovdiv). (2019) 61:240–248. doi: 10.2478/folmed-2018-0081 31301668

[51] Limon U Sezgin Akçay BI. Add-On Effect of Simultaneous Intravitreal Dexamethasone to Intravitreal Bevacizumab in Patients with Macular Edema Secondary to Branch Retinal Vein Occlusion. J Ocul Pharmacol Ther. (2022) 38:183–188. doi: 10.1089/jop.2021.0100 34964652

[52] Lucatto LFA Magalhães-Junior O Prazeres JMB Ferreira AM Oliveira RA Moraes NS . Incidence of anterior segment neovascularization during intravitreal treatment for macular edema secondary to central retinal vein occlusion. Arq Bras Oftalmol. (2017) 80:97–103. doi: 10.5935/0004-2749.20170024 28591282

[53] Meng L Yang M Jiang X Li Y Han X. Comparing ranibizumab, dexamethasone implant, and combined therapy for macular edema secondary to branch retinal vein occlusion: a clinical trial. Int Ophthalmol. (2024) 44:262. doi: 10.1007/s10792-024-03158-x 38913192

[54] Moon J Kim M Sagong M. Combination therapy of intravitreal bevacizumab with single simultaneous posterior subtenon triamcinolone acetonide for macular edema due to branch retinal vein occlusion. Eye (Lond). (2016) 30:1084–90. doi: 10.1038/eye.2016.96 27229707 PMC4985687

[55] Cekiç O Cakır M Yazıcı AT Alagöz N Bozkurt E Faruk Yılmaz O. A comparison of three different intravitreal treatment modalities of macular edema due to branch retinal vein occlusion. Curr Eye Res. (2010) 35:925–9. doi: 10.3109/02713683.2010.496540 20858114

[56] Rahman Irfanur Singh Ojaswita Kumari Pallavi Karak Pradeep. Visual and Anatomical Outcomes in AntiVEGF Versus Intravitreal Steroid Therapy for Macular Edema Secondary to Retinal Vein Occlusion: A Comparative Study.International Journal of Life Sciences, Biotechnology and Pharma Research. Vol. 14, (2025). doi: 10.69605/ijlbpr_14.2.2025.217

[57] Ramezani A Esfandiari H Entezari M Moradian S Soheilian M Dehsarvi B . Three intravitreal bevacizumab versus two intravitreal triamcinolone injections in recent-onset branch retinal vein occlusion. Graefes Arch Clin Exp Ophthalmol. (2012) 250:1149–60. doi: 10.1007/s00417-012-1941-8 22331147

[58] Ramezani A Esfandiari H Entezari M Moradian S Soheilian M Dehsarvi B . Three intravitreal bevacizumab versus two intravitreal triamcinolone injections in recent onset central retinal vein occlusion. Acta Ophthalmol. (2014) 92:e530–9. doi: 10.1111/aos.12317 24373344

[59] Higashiyama T Sawada O Kakinoki M Sawada T Kawamura H Ohji M. Prospective comparisons of intravitreal injections of triamcinolone acetonide and bevacizumab for macular oedema due to branch retinal vein occlusion. Acta Ophthalmol. (2013) 91:318–24. doi: 10.1111/j.1755-3768.2011.02298.x 22132711

[60] Wang HY Li X Wang YS Zhang ZF Li MH Su XN . Intravitreal injection of bevacizumab alone or with triamcinolone acetonide for treatment of macular edema caused by central retinal vein occlusion. Int J Ophthalmol. (2011) 4:89–94. doi: 10.3980/j.issn.2222-3959.2011.01.21 22553618 PMC3340676

[61] Ding X Li J Hu X Yu S Pan J Tang S. Prospective study of intravitreal triamcinolone acetonide versus bevacizumab for macular edema secondary to central retinal vein occlusion. Retina. (2011) 31:838–45. doi: 10.1097/IAE.0b013e3181f4420d 21293319

[62] Zhao M Zhang C Chen XM Teng Y Shi TW Liu F. Comparison of intravitreal injection of conbercept and triamcinolone acetonide for macular edema secondary to branch retinal vein occlusion. Int J Ophthalmol. (2020) 13:1765–1772. doi: 10.18240/ijo.2020.11.13 33215008 PMC7590870

