# Supplementary material for: The efficacy and safety of intraocular anti-VEGF injections versus anti-VEGF combined with steroids or steroid monotherapy for macular edema secondary to retinal vein occlusion: a systematic review and meta-analysis of randomized controlled trials
Source: Front Med (Lausanne). 2026 Jan 12;12:1727801. doi: 10.3389/fmed.2025.1727801 (PMC12832253; doi:10.3389/fmed.2025.1727801)
Supplement: Supplementary file 1 [file Data_Sheet_1.zip › Supplement Materials/Table 1 Baseline characteristics of studies and participants included.docx]

| **Author，year** | **NCT** | **No. of**  **participants** | **Disease** | **Steroids** | **Anti-VEGF** | **Combined therapy** |
| --- | --- | --- | --- | --- | --- | --- |
| Bandello 2018 | NCT01427751 | 307 | BRVO | DEX implant 154 | Ranibizumab 153 | NA |
| Cai 2024 | ChiCTR2400080048 | 44 | RVO | NA | Ranibizumab 23 | Ranibizumab+Dexamethason 21 |
| Campochiaro 2018 | NCT02303184 | 46 | RVO | NA | Aflibercept 23 | Aflibercept+CLS-TA 23 |
| Feltgen 2018 | NCT01580020 | 92 | BRVO | Dexamethasone 62 | Ranibizumab 113 | NA |
| Gado 2014 | NA | 60 | CRVO | Dexamethasone 30 | Ranibizumab 30 | NA |
| Ghader 2017 | TCTR20170612005 | 90 | CRVO | Triamcinolone 30 | Bevacizumab 30 | Triamcinolone+Bevacizumab 30 |
| Hattenbach 2018 | NCT01396057 | 244 | BRVO | Dexamethasone 118 | Ranibizumab 126 | NA |
| Hoerauf 2016 | NCT01396083 | 243 | CRVO | Dexamethason 119 | Ranibizumab 124 | NA |
| Kumar 2019 | NA | 30 | BRVO | Dexamethason 15 | Ranibizumab 15 | NA |
| Limon 2022 | NA | 67 | BRVO | NA | Bevacizumab 35 | Bevacizumab+Dexamethason 32 |
| Lucatto 2017 | NA | 35 | CRVO | Triamcinolone 11 | Bevacizumab 14 | NA |
| Maturi 2014 | NA | 30 | RVO | NA | Bevacizumab 15 | Bevacizumab+Dexamethason 15 |
| Meng 2024 | NA | 292 | BRVO | Dexamethason 98 | Ranibizumab 96 | Ranibizumab+Dexamethason 98 |
| Moon 2016 | NCT01614509 | 45 | BRVO | NA | Bevacizumab 23 | Bevacizumab+Triamcinolone 18 |
| Osman 2010 | NA | 52 | BRVO | Triamcinolone 17 | Bevacizumab 14 | Bevacizumab+Triamcinolone 21 |
| Rahman 2025 | NA | 100 | RVO | Dexamethasone 50 | Ranibizumab or  Aflibercept 50 | NA |
| Ramezani 2012 | NCT01044329 | 86 | BRVO | Dexamethasone 43 | Bevacizumab 43 | NA |
| Ramezani 2014 | NCT01178697 | 86 | CRVO | Triamcinolone 43 | Bevacizumab 43 | NA |
| Tomoaki 2013 | UMIN000001546 | 43 | BRVO | Triamcinolone 21 | Ranibizumab 22 | NA |
| Wang 2011 | NA | 75 | CRVO | NA | Bevacizumab 36 | Bevacizumab+Triamcinolone 39 |
| Xiao 2011 | NA | 31 | CRVO | Triamcinolone 16 | Bevacizumab 16 | NA |
| Zhao 2020 | ChiCTR1900028003 | 53 | BRVO | Triamcinolone 17 | Conbercept 36 | NA |

**Table1.Baseline characteristics of studies and participants included.**
