# Supplementary material for: The efficacy and safety of intraocular anti-VEGF injections versus anti-VEGF combined with steroids or steroid monotherapy for macular edema secondary to retinal vein occlusion: a systematic review and meta-analysis of randomized controlled trials
Source: Front Med (Lausanne). 2026 Jan 12;12:1727801. doi: 10.3389/fmed.2025.1727801 (PMC12832253; doi:10.3389/fmed.2025.1727801)
Supplement: Supplementary file 1 [file Data_Sheet_1.zip › Supplement Materials/search strategy.docx]

**From the default search time of the database to August 10, 2025.**

| PubMed | | |
| --- | --- | --- |
| No. | Query | Results |
| 1 | ((((((((((((((((vascular endothelial growth factor[Title/Abstract]) OR (VEGF[Title/Abstract])) OR (Bevacizumab[Title/Abstract])) OR (Avastin[Title/Abstract])) OR (Mvasi[Title/Abstract])) OR (Ranibizumab[Title/Abstract])) OR (RhuFab V2[Title/Abstract])) OR (V2, RhuFab[Title/Abstract])) OR (Lucentis[Title/Abstract])) OR (Aflibercept[Title/Abstract])) OR (eylea[Title/Abstract])) OR (Zaltrap[Title/Abstract])) OR (Pegaptanib[Title/Abstract])) OR (Macugen[Title/Abstract])) OR (pegaptanib sodium[Title/Abstract])) OR (conbercept[Title/Abstract])) OR (Brolucizumab[Title/Abstract]) | 130634 |
| 2 | (((((((((glucocorticoid[Title/Abstract]) OR (Steroids[Title/Abstract])) OR (Ozurdex[Title/Abstract])) OR (Yutiq[Title/Abstract])) OR (triam cinolone acetonide[Title/Abstract])) OR (dexamethasone[Title/Abstract])) OR (Triamcinolone acetonide[Title/Abstract])) OR (Triamcinolone[Title/Abstract])) OR (Nasacort[Title/Abstract])) OR (Azmacort[Title/Abstract]) | 235114 |
| 3 | (((((((((((((retinal vein occlusion[Title/Abstract]) OR (Occlusion, Retinal Vein[Title/Abstract])) OR (Retinal Vein Occlusions[Title/Abstract])) OR (Vein Occlusion, Retinal[Title/Abstract])) OR (Retinal Vein Thrombosis[Title/Abstract])) OR (Retinal Vein Thromboses[Title/Abstract])) OR (Vein Thrombosis, Retinal[Title/Abstract])) OR (Thrombosis, Retinal Vein[Title/Abstract])) OR (Branch Vein Occlusion[Title/Abstract])) OR (Occlusion, Branch Vein[Title/Abstract])) OR (Vein Occlusion, Branch[Title/Abstract])) OR (Branch Retinal Vein Occlusion[Title/Abstract])) OR (Retinal Branch Vein Occlusion[Title/Abstract])) OR (Central Retinal Vein Occlusion[Title/Abstract]) | 7036 |
| 4 | (((((((((((Macular edema[Title/Abstract]) OR (Edema, Macular[Title/Abstract])) OR (Macular Edema, Cystoid[Title/Abstract])) OR (Edema, Cystoid Macular[Title/Abstract])) OR (Cystoid Macular Dystrophy[Title/Abstract])) OR (Central Retinal Edema, Cystoid[Title/Abstract])) OR (Cystoid Macular Edema[Title/Abstract])) OR (Macular Dystrophy, Dominant Cystoid[Title/Abstract])) OR (Irvine-Gass Syndrome[Title/Abstract])) OR (Irvine Gass Syndrome[Title/Abstract])) OR (Syndrome, Irvine-Gass[Title/Abstract])) OR (Cystoid Macular Edema, Postoperativ[Title/Abstract]) | 14539 |
| 5 | (((((((Randomized Controlled Trial[Publication Type]) OR (randomized controlled trial[Title/Abstract])) OR (controlled clinical trial[Title/Abstract])) OR (randomized[Title/Abstract])) OR (placebo[Title/Abstract])) OR (clinical trials as topic[Title/Abstract])) OR (randomly[Title/Abstract])) OR (trial[Title/Abstract]) | 1821965 |
| 6 | #1 AND #2 AND #3 AND #4 AND #5 | 53 |
|  |  |  |

| Cochrane library | | |
| --- | --- | --- |
| No. | Query | Results |
| 1 | (vascular endothelial growth factor OR VEGF OR Bevacizumab OR Avastin OR Mvasi OR Ranibizumab OR RhuFab V2 OR V2, RhuFab OR Lucentis OR Aflibercept OR eylea OR Zaltrap OR Pegaptanib OR Macugen OR pegaptanib sodium OR conbercept OR Brolucizumab):ti,ab,kw | 16828 |
| 2 | (glucocorticoid OR Steroids OR Ozurdex OR Yutiq OR triam cinolone acetonide OR dexamethasone OR Triamcinolone acetonide OR Triamcinolone OR Nasacort OR Azmacort):ti,ab,kw | 36735 |
| 3 | (retinal vein occlusion OR Occlusion, Retinal Vein OR Retinal Vein Occlusions OR Vein Occlusion, Retinal OR Retinal Vein Thrombosis OR Retinal Vein Thromboses OR Vein Thrombosis, Retinal OR Thrombosis, Retinal Vein OR Branch Vein Occlusion OR Occlusion, Branch Vein OR Vein Occlusion, Branch OR Branch Retinal Vein Occlusion OR Retinal Branch Vein Occlusion OR Central Retinal Vein Occlusion):ti,ab,kw | 1119 |
| 4 | (Macular edema OR Edema, Macular OR Macular Edema, Cystoid OR Edema, Cystoid Macular OR Cystoid Macular Dystrophy OR Central Retinal Edema, Cystoid OR Cystoid Macular Edema OR Macular Dystrophy, Dominant Cystoid OR Irvine-Gass SyndromeOR Irvine Gass Syndrome OR Syndrome, Irvine-Gass OR Cystoid Macular Edema, Postoperativ):ti,ab,kw | 4192 |
| 5 | (randomized controlled tria OR controlled clinical trial OR randomized OR placebo OR clinical trials as topic OR randomly OR trial):ti,ab,kw | 1614886 |
| 6 | #1 AND #2 AND #3 AND #4 AND #5 | 85 |
|  |  |  |

| Web of science | | |
| --- | --- | --- |
| No. | Query | Results |
| 1 | TS=(vascular endothelial growth factor OR VEGF OR Bevacizumab OR Avastin OR Mvasi OR Ranibizumab OR RhuFab V2 OR V2, RhuFab OR Lucentis OR Aflibercept OR eylea OR Zaltrap OR Pegaptanib OR Macugen OR pegaptanib sodium OR conbercept OR Brolucizumab) | 191887 |
| 2 | TS=(glucocorticoid OR Steroids OR Ozurdex OR Yutiq OR triam cinolone acetonide OR dexamethasone OR Triamcinolone acetonide OR Triamcinolone OR Nasacort OR Azmacort) | 367667 |
| 3 | TS=(retinal vein occlusion OR Occlusion, Retinal Vein OR Retinal Vein Occlusions OR Vein Occlusion, Retinal OR Retinal Vein Thrombosis OR Retinal Vein Thromboses OR Vein Thrombosis, Retinal OR Thrombosis, Retinal Vein OR Branch Vein Occlusion OR Occlusion, Branch Vein OR Vein Occlusion, Branch OR Branch Retinal Vein Occlusion OR Retinal Branch Vein Occlusion OR Central Retinal Vein Occlusion) | 9363 |
| 4 | TS=(Macular edema OR Edema, Macular OR Macular Edema, Cystoid OR Edema, Cystoid Macular OR Cystoid Macular Dystrophy OR Central Retinal Edema, Cystoid OR Cystoid Macular Edema OR Macular Dystrophy, Dominant Cystoid OR Irvine-Gass SyndromeOR Irvine Gass Syndrome OR Syndrome, Irvine-Gass OR Cystoid Macular Edema, Postoperativ) | 20829 |
| 5 | TS=(randomized controlled tria OR controlled clinical trial OR randomized OR placebo OR clinical trials as topic OR randomly OR trial) | 3011320 |
| 6 | #1 AND #2 AND #3 AND #4 AND #5 | 226 |
|  |  |  |

| Embase | | |
| --- | --- | --- |
| No. | Query | Results |
| 1 | 'vascular endothelial growth factor':ab,ti OR 'VEGF':ab,ti OR 'Bevacizumab':ab,ti OR 'Avastin':ab,ti OR 'Mvasi':ab,ti OR 'Ranibizumab':ab,ti OR 'RhuFab V2':ab,ti OR 'V2, RhuFab':ab,ti OR 'Lucentis':ab,ti OR 'Aflibercept':ab,ti OR 'eylea':ab,ti OR 'Zaltrap':ab,ti OR 'Pegaptanib':ab,ti OR 'Macugen':ab,ti OR 'pegaptanib sodium':ab,ti OR conbercept:ab,ti OR Brolucizumab:ab,ti | 199001 |
| 2 | 'glucocorticoid':ab,ti OR 'Steroids':ab,ti OR 'Ozurdex':ab,ti OR 'Yutiq':ab,ti OR 'triam cinolone acetonide':ab,ti OR 'dexamethasone':ab,ti OR 'Triamcinolone acetonide':ab,ti OR 'Triamcinolone':ab,ti OR 'Nasacort':ab,ti OR 'Azmacort':ab,ti | 359938 |
| 3 | 'retinal vein occlusion':ab,ti OR 'Occlusion, Retinal Vein':ab,ti OR 'Retinal Vein Occlusions':ab,ti OR 'Vein Occlusion, Retinal':ab,ti OR 'Retinal Vein Thrombosis':ab,ti OR 'Retinal Vein Thromboses':ab,ti OR 'Vein Thrombosis, Retinal':ab,ti OR 'Thrombosis, Retinal Vein':ab,ti OR 'Branch Vein Occlusion':ab,ti OR 'Occlusion, Branch Vein':ab,ti OR 'Vein Occlusion, Branch':ab,ti OR 'Branch Retinal Vein Occlusion':ab,ti OR 'Retinal Branch Vein Occlusion':ab,ti OR 'Central Retinal Vein Occlusion':ab,ti | 9604 |
| 4 | 'Macular edema':ab,ti OR 'Edema, Macular':ab,ti OR 'Macular Edema, Cystoid':ab,ti OR 'Edema, Cystoid Macular':ab,ti OR 'Cystoid Macular Dystrophy':ab,ti OR 'Central Retinal Edema, Cystoid':ab,ti OR 'Cystoid Macular Edema':ab,ti OR 'Macular Dystrophy, Dominant Cystoid':ab,ti OR 'Irvine-Gass SyndromeOR Irvine Gass Syndrome':ab,ti OR 'Syndrome, Irvine-Gass':ab,ti OR 'Cystoid Macular Edema, Postoperativ':ab,ti | 20796 |
| 5 | 'randomized controlled trial':ab,ti OR 'controlled clinical trial':ab,ti OR randomized:ab,ti OR placebo:ab,ti OR 'clinical trials as topic':ab,ti OR randomly:ab,ti OR trial:ab,ti | 2714804 |
| 6 | #1 AND #2 AND #3 AND #4 AND #5 | 92 |
|  |  |  |
