# Supplementary figures and images for: The efficacy and safety of intraocular anti-VEGF injections versus anti-VEGF combined with steroids or steroid monotherapy for macular edema secondary to retinal vein occlusion: a systematic review and meta-analysis of randomized controlled trials
Source: Front Med (Lausanne). 2026 Jan 12;12:1727801. doi: 10.3389/fmed.2025.1727801 (PMC12832253; doi:10.3389/fmed.2025.1727801)

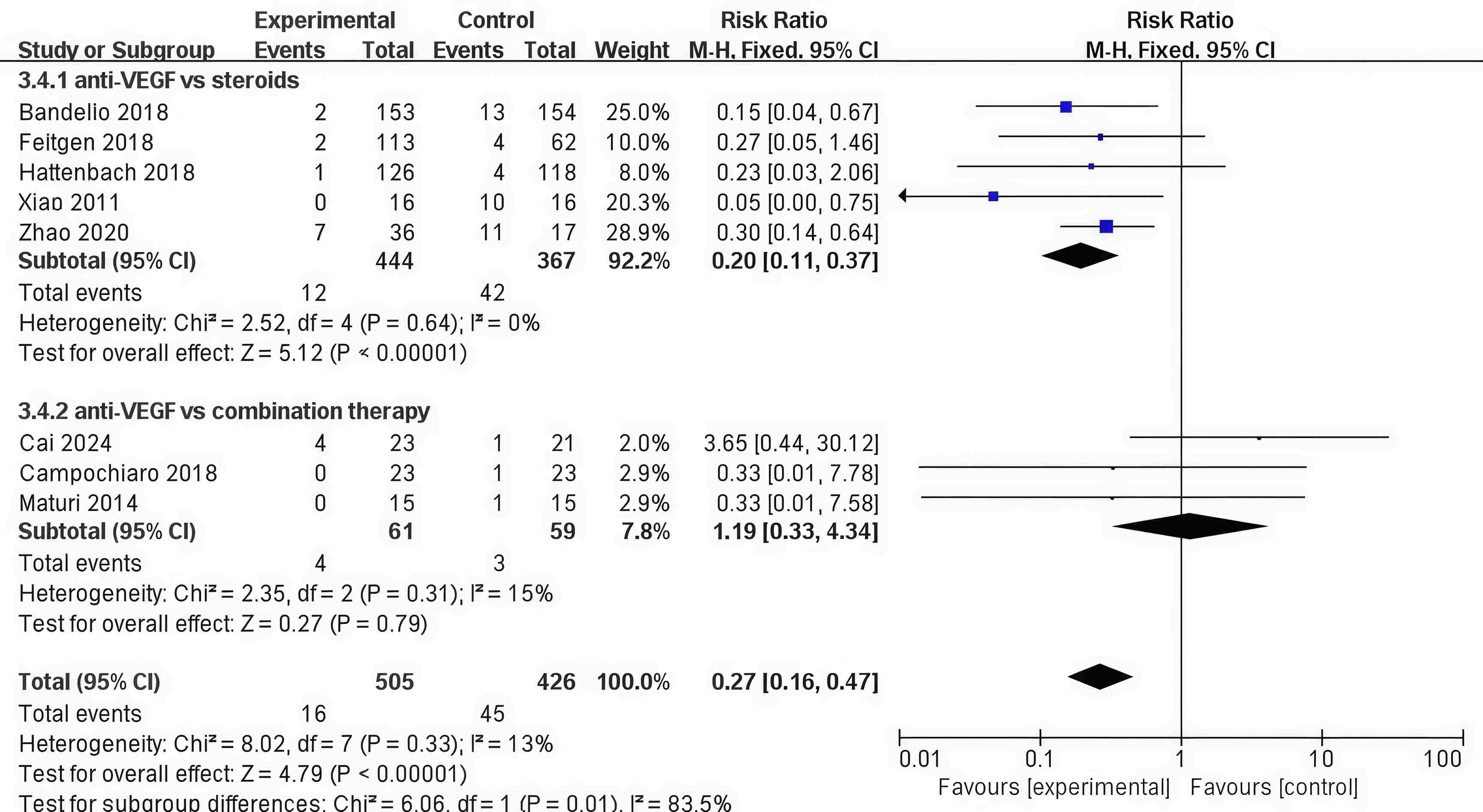

Supplement: Supplementary file 1 [file Data_Sheet_1.zip › Supplement Materials/Figure 1. Forest plot of the meta-analysis for cataract.jpeg]

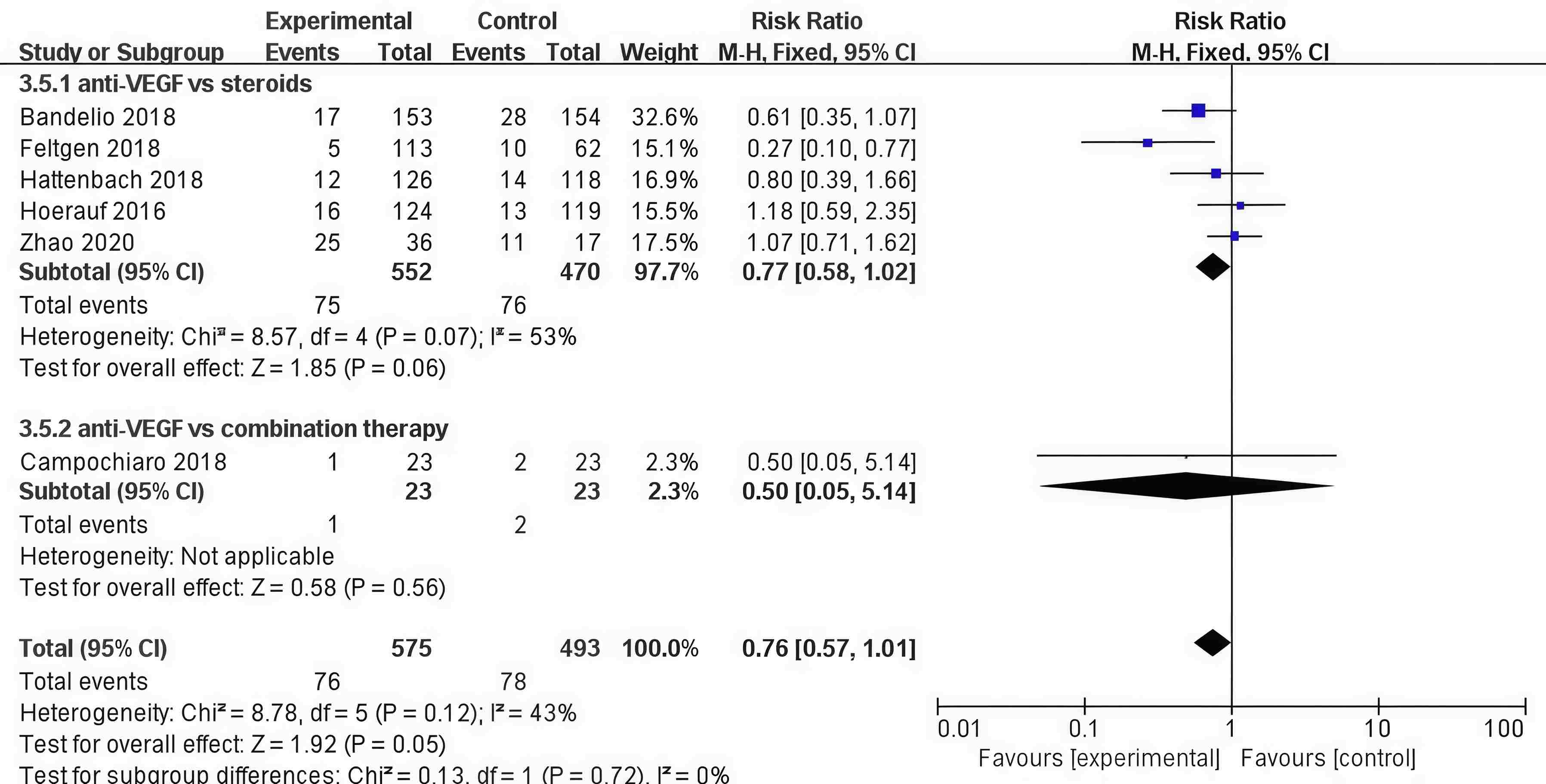

Supplement: Supplementary file 1 [file Data_Sheet_1.zip › Supplement Materials/Figure 2. Forest plot of the meta-analysis for conjunctival hemorrhage.jpeg]

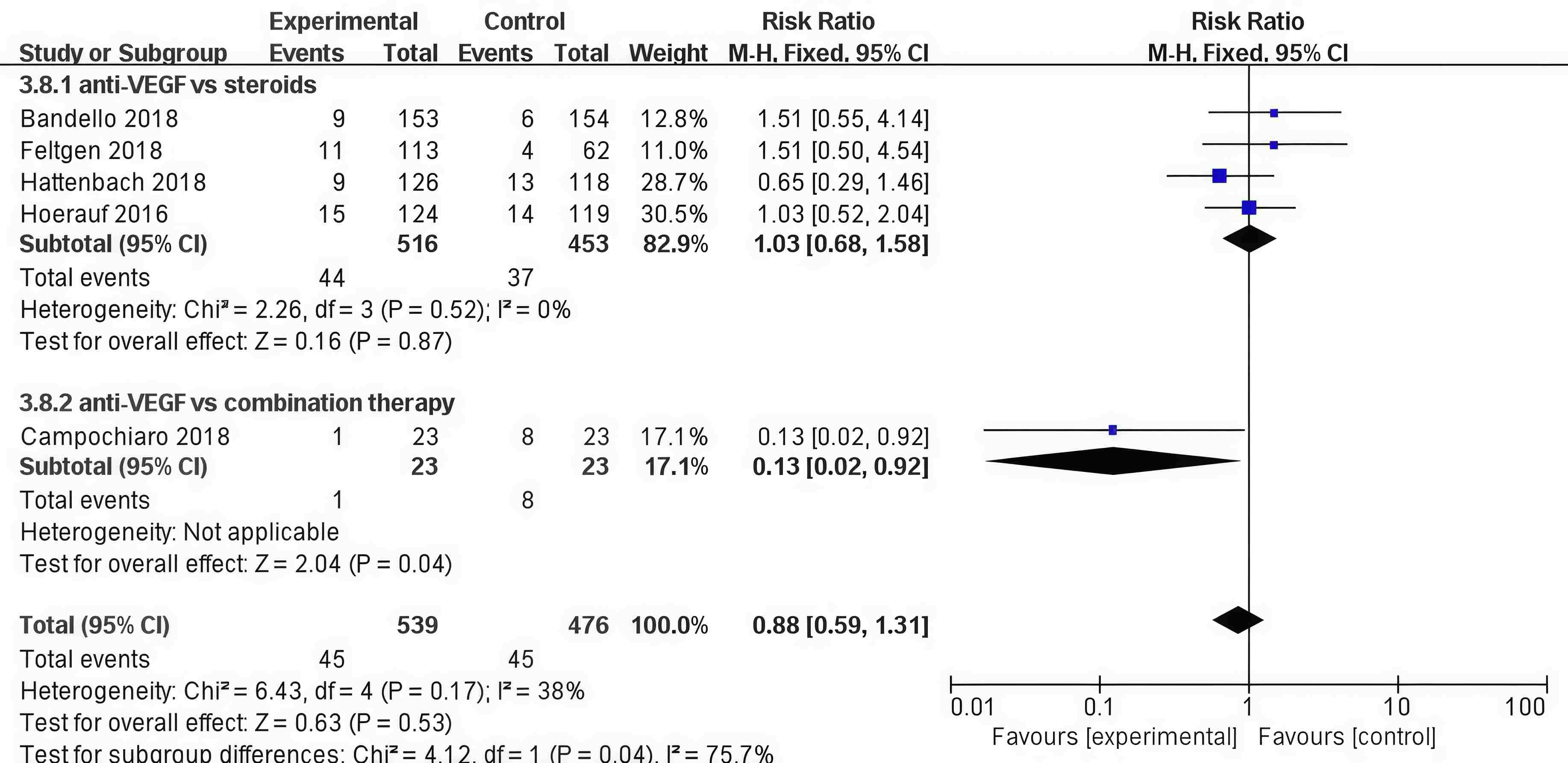

Supplement: Supplementary file 1 [file Data_Sheet_1.zip › Supplement Materials/Figure 3. Forest plot of the meta-analysis for eye pain.jpeg]

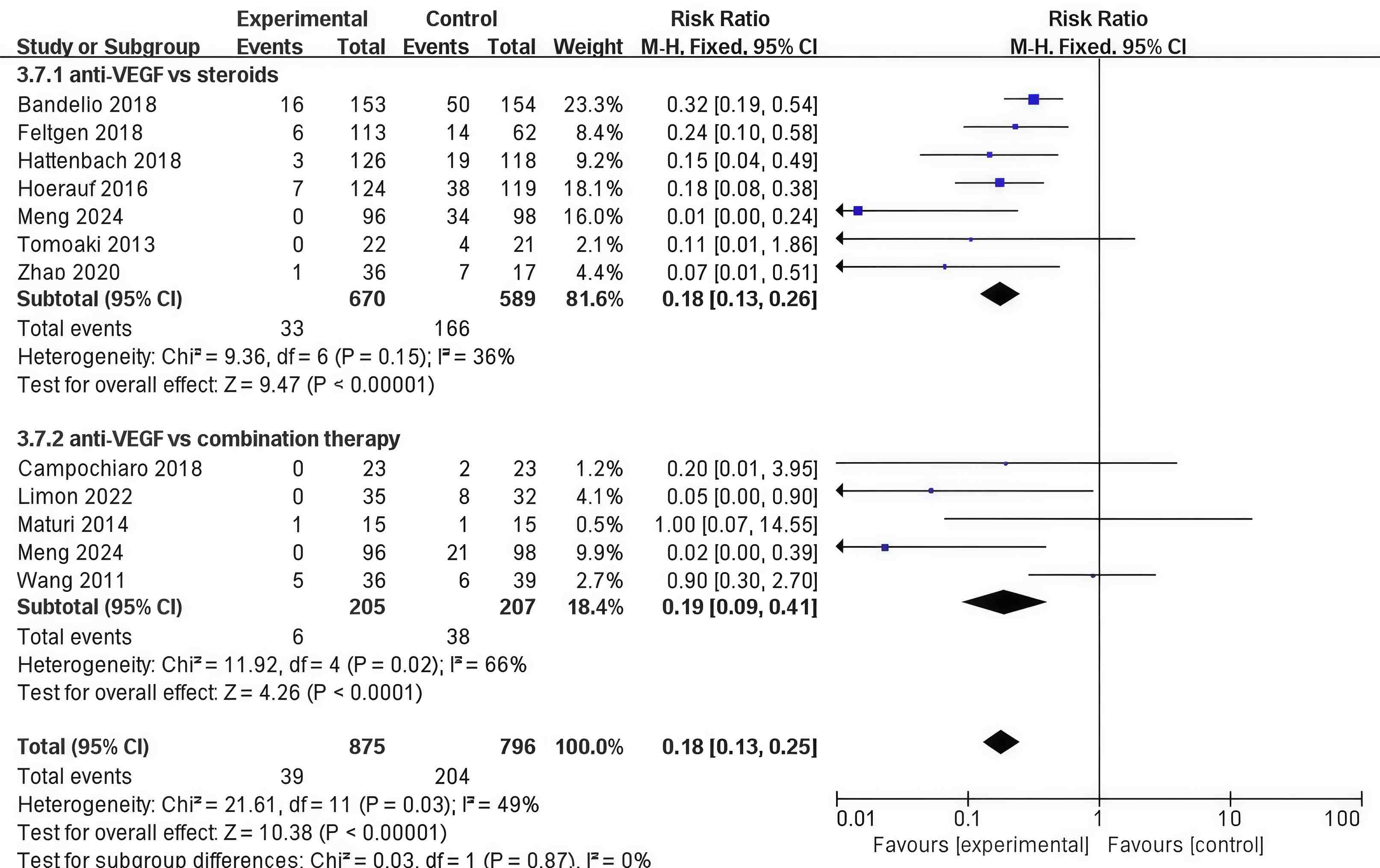

Supplement: Supplementary file 1 [file Data_Sheet_1.zip › Supplement Materials/Figure 4. Forest plot of the meta-analysis for intraocular pressure.jpeg]

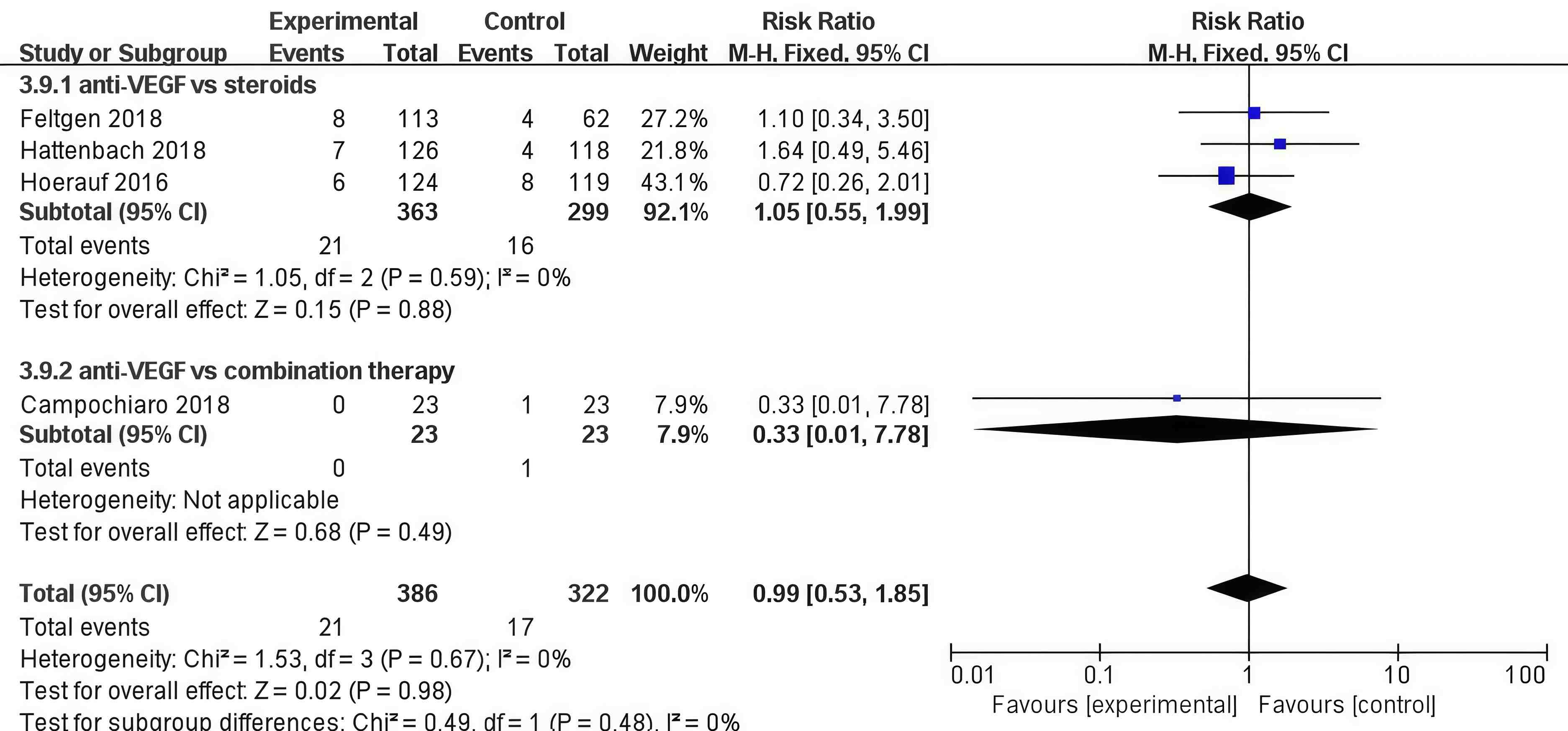

Supplement: Supplementary file 1 [file Data_Sheet_1.zip › Supplement Materials/Figure 5. Forest plot of the meta-analysis for lacrimation increased.jpeg]

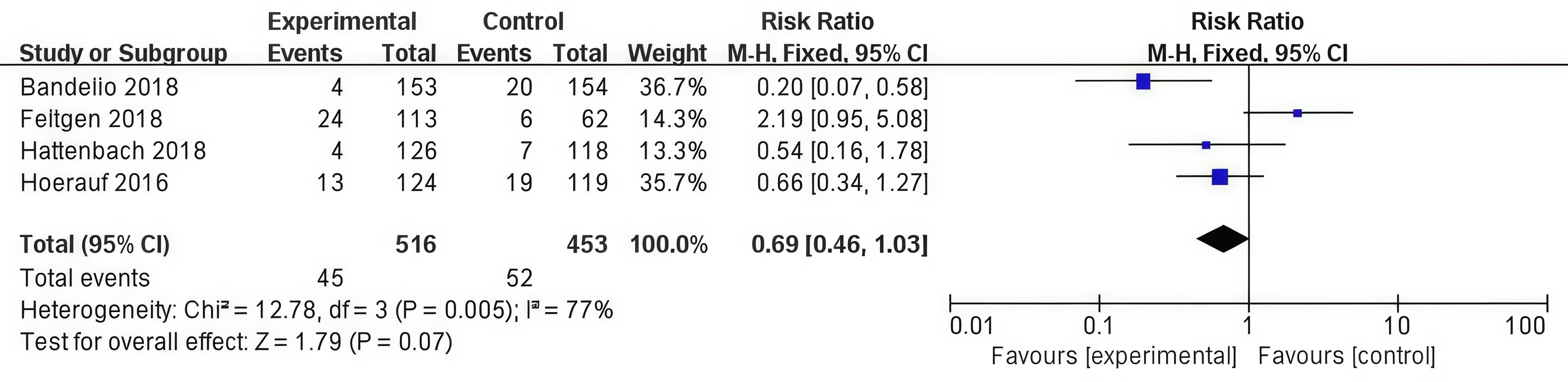

Supplement: Supplementary file 1 [file Data_Sheet_1.zip › Supplement Materials/Figure 6. Forest plot of the meta-analysis for macular edema.jpeg]

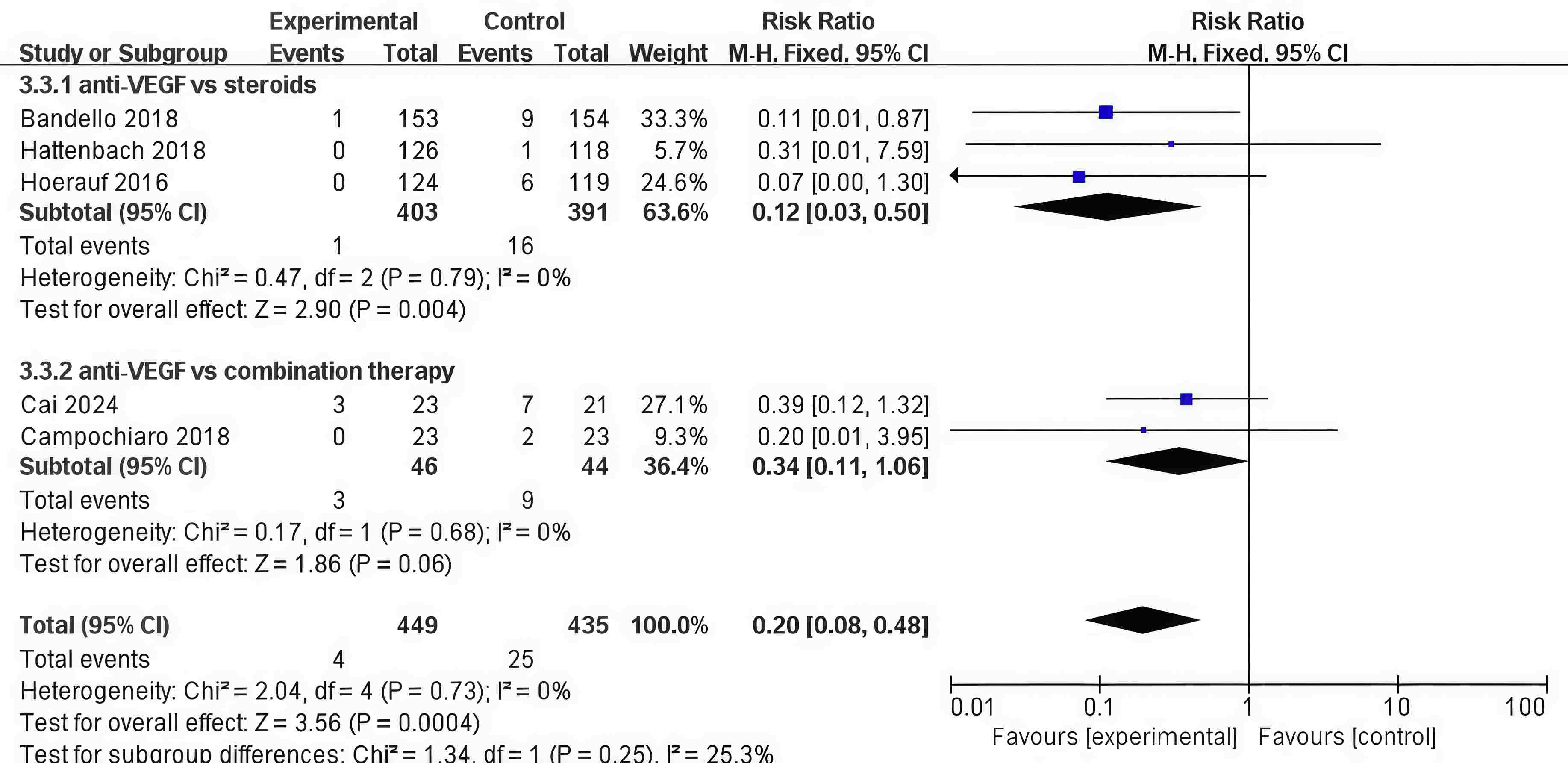

Supplement: Supplementary file 1 [file Data_Sheet_1.zip › Supplement Materials/Figure 7. Forest plot of the meta-analysis for ocular hypertension.jpeg]

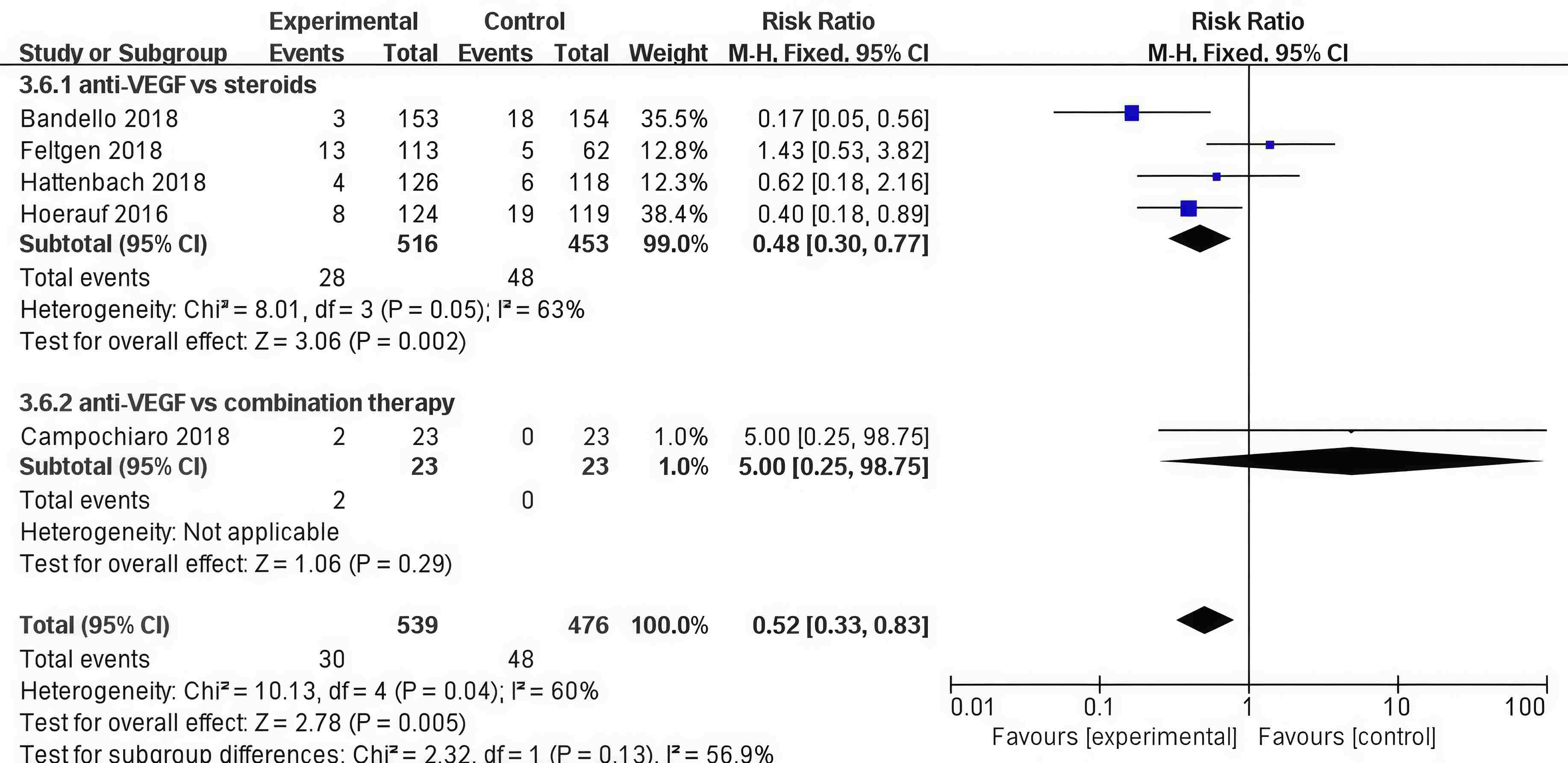

Supplement: Supplementary file 1 [file Data_Sheet_1.zip › Supplement Materials/Figure 8. Forest plot of the meta-analysis for reduced visual acuity.jpeg]
